# Synthetic silica fibers of different length, diameter and shape: synthesis and interaction with rat (NR8383) and human (THP-1) macrophages in vitro, including chemotaxis and gene expression profile

**DOI:** 10.1186/s12989-024-00586-6

**Published:** 2024-05-11

**Authors:** Nataniel Białas, Nina Rosenkranz, Daniel Gilbert Weber, Kathrin Kostka, Georg Johnen, Aileen Winter, Alexander Brik, Kateryna Loza, Katja Szafranski, Thomas Brüning, Jürgen Bünger, Götz Westphal, Matthias Epple

**Affiliations:** 1 Inorganic Chemistry and Center for Nanointegration Duisburg-Essen (CENIDE), University of Duisburg-Essen, 45141 Essen, Germany; 2 Institute for Prevention and Occupational Medicine of the German Social Accident Insurance, Institute of the Ruhr University Bochum (IPA), 44789 Bochum, Germany

## Abstract

**Background:**

Inhalation of biopersistent fibers like asbestos can cause strong chronic inflammatory effects, often resulting in fibrosis or even cancer. The interplay between fiber shape, fiber size and the resulting biological effects is still poorly understood due to the lack of reference materials.

**Results:**

We investigated how length, diameter, aspect ratio, and shape of synthetic silica fibers influence inflammatory effects at doses up to 250 µg cm^-2^. Silica nanofibers were prepared with different diameter and shape. Straight (length ca. 6 to 8 µm, thickness ca. 0.25 to 0.35 µm, aspect ratio ca. 17:1 to 32:1) and curly fibers (length ca. 9 µm, thickness ca. 0.13 µm, radius of curvature ca. 0.5 µm, aspect ratio ca. 70:1) were dispersed in water with no apparent change in the fiber shape during up to 28 days. Upon immersion in aqueous saline (DPBS), the fibers released about 5 wt% silica after 7 days irrespectively of their shape. The uptake of the fibers by macrophages (human THP-1 and rat NR8383) was studied by scanning electron microscopy and confocal laser scanning microscopy. Some fibers were completely taken up whereas others were only partially internalized, leading to visual damage of the cell wall. The biological effects were assessed by determining cell toxicity, particle-induced chemotaxis, and the induction of gene expression of inflammatory mediators.

**Conclusions:**

Straight fibers were only slightly cytotoxic and caused weak cell migration, regardless of their thickness, while the curly fibers were more toxic and caused significantly stronger chemotaxis. Curly fibers also had the strongest effect on the expression of cytokines and chemokines. This may be due to the different aspect ratio or its twisted shape.

**Supplementary Information:**

The online version contains supplementary material available at 10.1186/s12989-024-00586-6.

## Introduction

The WHO defines fibers as particles with an aspect ratio greater than 3:1, a length greater than 5 µm, and a thickness less than 3 µm in diameter [1–3]. Evidence increases that this definition does not completely explain the observed biological effects [4]. Prolonged inhalation of biopersistent fibers leads to chronic inflammation of the airways which is supposed to induce fibrosis and even cancer. However, the causality in humans has been proven only for asbestos [5]. Other biopersistent fibers were classified based on animal testing and analogy conclusion [6]. Nevertheless, in the interest of precautionary health protection, it is mandatory to assume that fibers that have a carcinogenic effect in animal experiments are also carcinogenic in humans. In fact, some newly developed advanced materials like multiwalled carbon nanotubes (MWCNT) are strongly carcinogenic in experimental animal studies. Rittinghausen and co-workers demonstrated strong carcinogenic effects of four different types of MWCNT. Besides aspect ratio, curvature seems to be an important parameter influencing the carcinogenicity of MWCNT [7]. Nagai et al. proposed that the small diameter and the needle-like shape of MWCNT contribute to their strong toxicity [8]. Thus, there is increasing evidence that additional properties of biopersistent fibers contribute to their toxicity besides length and thickness.

Silicates form the chemical basis of many naturally occurring and synthetic fibers, e.g., mineral wool [1, 3, 9–12]. To analyze the toxicity of fiber-induced inflammation at the cellular level, the use of synthetic silica fibers with defined length, diameter, and shape is useful [13], considering also the fact that they do not exert an inherent toxicity [14]. The intensity of inflammation is expressed by the number of inflammatory cells that are attracted to the inflamed loci. In particular, the number of migrated neutrophilic granulocytes determines the severity of the inflammation. This can be assessed by the particle-induced cell migration assay (PICMA). This assay uses supernatants from particle-challenged rat alveolar macrophages NR8383 as chemoattractants for differentiated HL-60 cells [15]. Granular biopersistent particles (GBP) such as amorphous silica (hydrated silicon dioxide), quartz, or carbon black induced marked chemotaxis in this assay whereas inert particles like barium sulfate had no such effect [16]. In addition, much stronger chemotaxis was caused by asbestos fibers and even more by MWCNT compared to GBP [16]. This reflects the strong toxicity of asbestos and MWCNT *in vivo* [3, 11]. Thus, PICMA is a validated tool for comparative *in vitro* experiments as performed in our study.

This study aims at the identification of characteristics that influence fiber toxicity using synthetic silica fibers of different length, diameter, and shape. After developing a robust synthesis, the interaction of such well-defined fibers with macrophages and the resulting cell-biological effects were elucidated in detail.

## Materials and methods

### Reagents for fiber synthesis

We used the following chemicals for synthesis and preparation of silica fibers: Poly(N-vinylpyrrolidone) (PVP; *M*_w_ 55,000 g mol^-1^; Sigma-Aldrich, USA), 1-pentanol (98%; Carl Roth, Germany), ethanol (99%; Fisher Chemicals, USA), sodium citrate dihydrate (99%; Carl Roth), aqueous ammonia solution (30-33%; Carl Roth), tetraethoxysilane (TEOS; 98%; Sigma-Aldrich), and sodium hydroxide (Fisher Chemicals). UltraPure sterile nuclease-free water (Invitrogen, USA) was used for all fiber syntheses to ensure the microbiological purity of the samples, which was a necessary criterion for all biological studies. Autoclaved pure water (ELGA Purelab, UK) was used for all other purposes. Sterile disposable laboratory materials (Sarstedt, Germany) and chemically purified, heat-sterilized, and depyrogenated (250 °C, 1 h) laboratory glassware were used for syntheses and handling of the samples. The silica fibers and the reference samples were dispersed in Dulbecco's phosphate-buffered saline (DPBS; Gibco, USA) for solubility studies.

### Methods and instruments for silica fiber synthesis and characterization

Scanning electron microscopy (SEM) was performed with an Apreo S LoVac instrument (Thermo Fisher Scientific, USA). Prior to analysis, all samples (including cells) were sputtered with a conducting gold/palladium coating. Energy-dispersive X-ray spectroscopy (EDX) was performed with an UltraDry silicon drift X-ray detector (Thermo Scientific). X-ray powder diffraction (XRD) was carried out on a D8 Advance instrument (Bruker, USA) in Bragg-Brentano mode with Cu Kα radiation (1.54 Å; 40 kV; 40 mA). The release of silicate was determined by inductively-coupled plasma mass spectrometry (ICP-MS) with a 7900 ICP-MS device (Agilent, USA) at Mikrolab Kolbe (Germany). Centrifugation of the fiber samples was carried out at room temperature with a Heraeus Multifuge X1R instrument (Thermo Scientific). The fiber pellets were redispersed with an S10 Elmasonic ultrasound bath (Elma, Germany) and mixed, where appropriate, with a Vortex-Genie 2 instrument (Scientific Industries, USA). For sample drying, as well as heat sterilization of synthesis glassware, a UF110 laboratory oven was used (Memmert, Germany). All fiber samples were prepared and stored at ambient temperature before the subsequent biological tests.

### Silica fiber synthesis

The synthesis of silica fibers in a wet-chemical approach (water-in-oil microemulsion) was performed according to Kuijk et al. [17] with some modifications [13]. Prior to use, round-bottom flasks were chemically purified first with boiling 1 M NaOH and then twice with boiling water. Next, the flasks were dried, heat-sterilized, and depyrogenated.

To synthesize straight thick silica fibers, 3 g PVP were dissolved in 30 mL 1-pentanol by vigorous stirring in a 500 mL round-bottom flask. Next, 3 mL ethanol, 0.84 mL water, 0.2 mL 0.18 M sodium citrate dihydrate, and 0.68 mL aqueous ammonia solution (25-30%) were added to the reaction mixture, which was then stirred for 20 min. After the stirring was stopped, the stir bar was removed, and 0.3 mL TEOS was added dropwise to the reaction mixture. The flask was gently shaken manually for 30 s and left at ambient temperature for 16 h. The fibers were collected by centrifugation (4,000 rpm, 3,000 x g, 30 min). The fiber pellet was redispersed and washed twice with 30 mL ethanol. The fiber sample was dried in air at 80 °C for 5 h. The yield was determined gravimetrically. A typical synthesis of straight thick silica fibers gave 30-40 mg with good reproducibility.

In order to synthesize straight thin silica fibers, the same amounts of reagents were mixed as for the synthesis of straight thick silica fibers. The only modification of the synthesis procedure was that, prior to the addition of TEOS to the reaction mixture, the round-bottom flask containing the reaction mixture was placed in an ultrasound bath and ultrasonicated for 30 s to decrease the water droplet size in the emulsion. Water droplets serve as microreactors for TEOS nucleation in the microemulsion synthesis. Further handling of the straight thin fiber sample was performed as with straight thick silica fibers. A typical synthesis of straight thin silica fibers gave 30-40 mg with good reproducibility.

Finally, to synthesize thin curly silica fibers, the protocol for the synthesis of straight thick silica fibers was modified, i.e. instead of 3 mL ethanol, 6 mL of ethanol were added to the reaction mixture. This increased the ethanol-to-water ratio in the synthesis from 1.75:1 to 3.5:1 (*v*/*v*) and led to curly silica fibers. The amounts of the other reagents in the synthesis were not changed, and the reaction mixture was not ultrasonicated. Further handling of the curly fiber sample was performed as with the straight silica fibers. A typical synthesis of the curly silica fibers gave about 10-15 mg with good reproducibility.

### Release of silica upon immersion in DPBS

1 mg mL^-1^ dispersions of the silica fibers with different shape (straight thick, straight thin, and curly), as well as spherical silica nanoparticles (as reference; 10-20 nm diameter, unfunctionalized; Sigma-Aldrich) and microcrystalline quartz (as reference; Sikron^®^ SH500; Quarzwerke, Germany) were prepared in DPBS, thoroughly vortexed and stored for 7 days at room temperature without stirring to avoid mechanical damage of the fibers. After incubation, all particulate material was removed by spin filtration (10,000 MWCO; Merck Millipore, Germany). The concentration of released silicon in the particle-free filtrates was determined by ICP-MS.

### Immersion stability studies on silica fibers

Long-term stability experiments were performed with 1 mg mL^-1^ silica fiber dispersions in water. The dispersions were thoroughly vortexed and stored for 7, 14, 21, and 28 days at ambient temperature without stirring to avoid mechanical damage of the fibers. After each incubation time point, the water phase above the fiber pellet was gently removed and a fiber sample was taken for SEM analysis.

### Uptake of silica fibers by THP-1 cells investigated by scanning electron microscopy

Glass microscopy slides (Sarstedt) in 24-well plates were first coated with poly-L-lysine (PLL) for 1 h at 37 °C and 5% CO_2_ atmosphere. THP-1 cells (monocytes/macrophages, human acute monocytic leukemia cell line; ATCC TIB-202, LGC Standards, Germany) were cultivated in Roswell Park Memorial Institute medium (RPMI; Gibco, Invitrogen, Germany) with 10% FBS (37 °C, 5% CO_2_ atmosphere) for 48 h and then differentiated with phorbol 12-myristate 13-acetate (PMA, 100 nM; P1585, Sigma-Aldrich) on the prepared glass microscopy slides (2·10^5^ cells per well). After 72 h, the cells were incubated with straight or curly silica fibers (12 µg cm^-2^, 24 µg cm^-2^, 48 µg cm^-2^, 96 µg cm^-2^) for 24 h. For fixation, the cells were treated for 15 min at room temperature with 3.7% glutaraldehyde solution (Sigma-Aldrich), washed three times with phosphate-buffered saline (PBS; Gibco) and dehydrated with an ascending ethanol row (20%, 40%, 60%, 80% and 96%) for 5 min for each sequence. Finally, the cells were left for 2 h to dry in air.

### Uptake of silica fibers by NR8383 cells investigated by scanning electron microscopy

Glass microscopy slides (Sarstedt) in 24-well plates were first coated with poly-L-lysine (PLL) for 1 h at 37 °C and 5% CO_2_ atmosphere. NR8383 rat macrophages (3∙10^6^ cells mL^-1^) were suspended in 1 mL Ham's F12 medium containing 15% FCS, 2 mM L-glutamine, 100 µg mL^-1^ penicillin, and 100 U mL^-1^ streptomycin. Culture medium was added to a final volume of 3 mL (equivalent to 2.4⋅10^5^ cells cm^-2^; area of well: 3.5 cm^2^). The fibers were dispersed in 1 mL culture medium and added to the cells. The cells were incubated with straight and curly silica fibers (12 µg cm^-2^, 24 µg cm^-2^, 48 µg cm^-2^, and 96 µg cm^-2^) for 16 h. Untreated cells served as negative control. Incubation of the cells with the fibers was performed at 37 °C at 100% humidity and 5% CO_2_ for 16 h. For fixation, the cells were treated for 15 min at room temperature with 3.7% glutaraldehyde (Sigma-Aldrich), washed three times with PBS, and dehydrated with an ascending ethanol row (20%, 40%, 60%, 80% and 96%) for 5 min for each sequence. Finally, the cells were left for 2 h to dry in air. Cross-sections were prepared by freeze-fracturing of the glass microscopy slides after shock-immersion in liquid nitrogen.

### Uptake of silica fibers by THP-1 cells investigated by confocal laser scanning microscopy (CLSM)

THP-1 cells were cultivated in RPMI medium with 10% fetal bovine serum (FBS; Gibco) at 37 °C and 5% CO_2_ atmosphere for 48 h in cell culture flasks. Cells were then seeded onto 8-well plates (2·10^5^ cells per well; area of well: 1 cm^2^), exposed to PMA (100 nM) for differentiation, and cultivated for 72 h. In the next step, the cells were incubated with straight or curly silica fibers (12 µg cm^-2^, 24 µg cm^-2^, 48 µg cm^-2^, 96 µg cm^-2^) for 24 h. The fibers were fluorescently labelled before the incubation by stirring the fiber dispersion in ethanol (2 mg mL^-1^) at room temperature in darkness overnight in the presence of 4 µL PEI-rhodamine (PEI: polyethyleneimine; 1 mg mL^-1^, *M*_*w*_ = 25,000 g mol^-1^, Surflay, Germany) that adsorbed on the fiber surface. Purification of the fibers was performed by triple centrifugation (3,500 rpm, 2,300 x g, 10 min) in ethanol, indicating a strong adhesion of the labelled polyelectrolyte to the fibers, followed by lyophilization.

The cells were fixed with a 3.7% paraformaldehyde (PFA) solution at ambient temperature for 10 min. After washing three times with PBS the cell membrane was stained with AlexaFluor-647-phalloidin (Invitrogen, Germany) for 20 min in the incubator (37 °C and 5% CO_2_ atmosphere). After washing three times with PBS, the nuclei were stained with Hoechst3342 (Invitrogen) for 15 min. Finally, the cells were washed again in PBS and then stored in PBS. Confocal laser scanning microscopy was carried out with a Leica TCS SP8X FALCON instrument.

### Microbial*** purity***

Microbial purity of samples was measured with an Endosafe^®^ nexgen-PTS™ spectrophotometer (Charles River, USA) with disposable cartridges (PTS2001F, Charles River) and an endotoxin detection range of 0.01-1 EU mL^-1^. The microbial purity of the samples was assessed by the limulus amebocyte lysate (LAL) chromogenic assay, based on the detection of endotoxins which are part of the lipopolysaccharide membrane of Gram-negative bacteria. All samples were below 0.1 EU mL^-1^. Thus, all fibers were considered as pyrogen-free for the subsequent biological studies with fiber exposure. This is a major prerequisite for valid biological studies [18].

### Cell viability assay (MTT) with silica fiber-incubated THP-1 cells

A viability assay with 3-(4,5-dimethylthiazol-2-yl)-2,5-diphenyltetrazolium bromide (MTT; Invitrogen) was performed to investigate the viability of THP-1 cells. After culturing for 24 h, the cells were transferred to a 24-well plate (5·10^4^ cells per well; area of well: 1.9 cm^2^) in 500 µL RPMI and differentiated for 72 h with PMA. In the next step, the cells were incubated with straight or curly fibers (2.6 µg cm^-2^, 13 µg cm^-2^, 26 µg cm^-2^) for 24 h.

For the MTT assay, the cells were washed three times with PBS and incubated with 300 µL MTT solution (1 g L^-1^) for 1 h at 37 °C in 5% CO_2_ atmosphere. Subsequently, the MTT solution was replaced by 300 µL dimethyl sulfoxide (DMSO; Sigma-Aldrich) for another 30 min. Finally, triplicate samples of the DMSO solution were transferred to a 96-well plate (100 µL aliquots per well) for spectrophotometric analysis at 570 nm. A control group with untreated cells (mock) served as reference for the relative cell viability.

### Viability assay (AlamarBlue) with silica fiber-incubated NR8383 cells

The cell viability was investigated with the AlamarBlue Assay (Invitrogen). Approximately 10^4^ NR8383 rat macrophages in 100 µL full growth medium (Ham's F12 medium containing 15% FCS, 2 mM L-glutamine, 100 µg mL^-1^ penicillin, and 100 U mL^-1^ streptomycin) were seeded into 96-well fluorescence cell culture plates (Corning, USA; area of well: 0.285 cm^2^). The cells were incubated at 37 °C at 100% humidity and 5% CO_2_ for 24 h. The fibers were suspended in 100 µL full growth medium at a maximum fiber concentration of 512 µg cm^-2^, followed by 1:2 dilution steps to the desired final concentration, and incubated with the macrophages for 24 h. Next, 22 µL of the cell viability reagent of the assay were added. After another 2 h of incubation, the viability was measured with a fluorescence plate reader at 560/590 nm (SpectraMax M3, Molecular Devices, USA). Three independent experiments were carried out. All particle dispersions were used, stored, and treated identically to reduce experimental variance.

### Differentiation of HL-60 cells

HL-60 cells were obtained from DSMZ (Braunschweig, Germany). For the investigation of the chemotaxis, we used trans-retinal-differentiated HL-60 cells (dHL-60). The HL-60 cells were cultivated in RPMI 1640 medium (PAN-Biotech, Germany), 10% FSC, 2 mM L-glutamine, 100 μg mL^-1^ penicillin, 100 U mL^-1^ streptomycin, and 1 μM trans-retinal at 37 °C, 100% humidity and 5% CO_2_, for three days as described earlier [19].

### Incubation of NR8383 cells with silica fibers and PICMA

NR8383 rat macrophages (3∙10^6^ cells mL^-1^) were suspended in 1 mL Ham's F12 medium containing 15% FCS, 2 mM L-glutamine, 100 µg mL^-1^ penicillin, and 100 U mL^-1^ streptomycin. Culture medium was added to a final volume of 3 mL (equivalent to 2.4⋅10^5^ cells cm^-2^; area of flask: 12.5 cm^2^). The fibers were dispersed in 1 mL culture medium at a maximum concentration of 96 µg cm^-2^, followed by dilution and added to the cells. Cells without fibers served as negative control. Incubation of the cells with the fibers was performed at 37 °C, 100% humidity, and 5% CO_2_ for 16 h. Thereafter, the cells were transferred to 5 mL Eppendorf vials. Cells were removed by centrifugation at 400 g for 5 min. The fibers were removed by centrifugation at 15,000 g for 10 min. 500 µL of the supernatants were immediately used for PICMA. The remaining supernatant was stored until further use at -18 °C. For RNA stabilization, approximately 5⋅10^6^ cells were collected in 500 µL RNAlater (Thermo Scientific), stored at 4 °C overnight and subsequently at -20°C until RNA isolation and analysis.

### Chemotaxis Assay (PICMA)

Cell migration was investigated according to Boyden [20] with the modifications described in refs. [15, 16]. 2⋅10^5^ unchallenged dHL-60 cells in RPMI 1640 medium without FCS were seeded in a plate well insert (THINCERT, 3 µm pore size, Greiner bio-one, Germany) that was placed in the cavities of 24-black well plates (Krystal, Dunn Labortechnik, Germany). 500 µL of the supernatants of the particle-challenged macrophages were added to the lower chamber. Migration of dHL-60 cells across the membrane was performed at 37 °C, 100% humidity and 5% CO_2_ for 24 h. For calibration, 0 to 10^5^ HL-60 cells were seeded directly into four-well plates that were left without inserts.

Staining of the cells was performed by adding 500 µL calcein-AM (>90% by HPLC, Sigma-Aldrich) for 60 min at 37 °C, 100% humidity and 5% CO_2_. The cell suspensions were removed from the plate wells and collected at 400 g for 5 min at room temperature. 850 µL of the supernatant were discarded while the cells were resuspended in the remaining volume of 150 µL. In addition, the adherent cells at the outside of the inserts were detached by adding 500 µL trypsin/EDTA (0.05%/0.02%, PAN-Biotech) for 10 min at 37 °C, 100% humidity and 5% CO_2_. Subsequently, the inserts were removed from the plate wells. Then 150 µL of the collected cells were added into the plate wells that contained the 500 µL of the trypsin/EDTA detached cells. The cell number was determined by fluorescence measurement at 490/520 nm and calculated from the cell calibration (SpectraMax M3, Molecular Devices).

Two consecutive tests series were carried out with three independently conducted experiments each. For each test set, the synthetic silicate fibers were dispersed in parallel, and for both experimental sets the same time regime was maintained.

EC50 and IC50 values of the chemotaxis- and AlamarBlue assays were calculated by non-linear regression analysis. The differences between the slopes of the chemotaxis and viability curves were calculated based on the linear courses of the slopes with a linear regression. All statistical calculations were done with GraphPad Prism 10.1.2.

### RNA isolation

RNA was isolated with the RNeasy Plus kit (Qiagen, Germany) according to the manufacturer's instruction. The RNA was quantified by measuring the absorbance with a NanoDrop ND-100 spectrophotometer (Thermo Scientific). The integrity of the isolated RNA was measured with a 2100 Bioanalyzer with RNA 6000 Nano Kits (Agilent). RNA concentration, absorbance, and RNA integrity number (RIN) are all given in Supplementary Table S1.

### RNA expression analysis

QuantiNova LNA PCR Focus Panel Rat Cytokines & Chemokines assays (GeneGlobe ID: SBRN-150Z; Qiagen) were used for expression analysis of 84 rat chemokines and cytokines with a 7900 HT Fast Real-Time PCR System (Thermo Scientific) according to the manufacturer's instruction for a two-step RT-qPCR (reverse transcription quantitative polymerase chain reaction). For RT, 0.5 µg RNA was used as template. Additionally, 1 µL of Internal Control RNA (Thermo Scientific) was used as quality control. Subsequently, 90 µL H_2_O was added to the reaction product, and 100 µL of this mixture was used as template for PCR. Quality controls and exclusion of genomic DNA were ensured with the internal controls of the panels. All experiments were done in triplicates. Expression analysis was performed with the QuantiNova LNA PCR Data Analysis Software (Qiagen) with Student's *t*-test. Cut-off parameters for statistically significant altered expression were fold change (FC) >2.0 (upregulation) or <0.5 (downregulation) [21] and *p*<0.05. For identification of the most suitable references, the web-based comprehensive tool RefFinder [22] was used to analyze the five included candidate references *Actb*, *B2m*, *Hprt1*, *Ldha*, and *Rplp1*.

## Results

### Synthesis of silica fibers

**Fig. 1:**
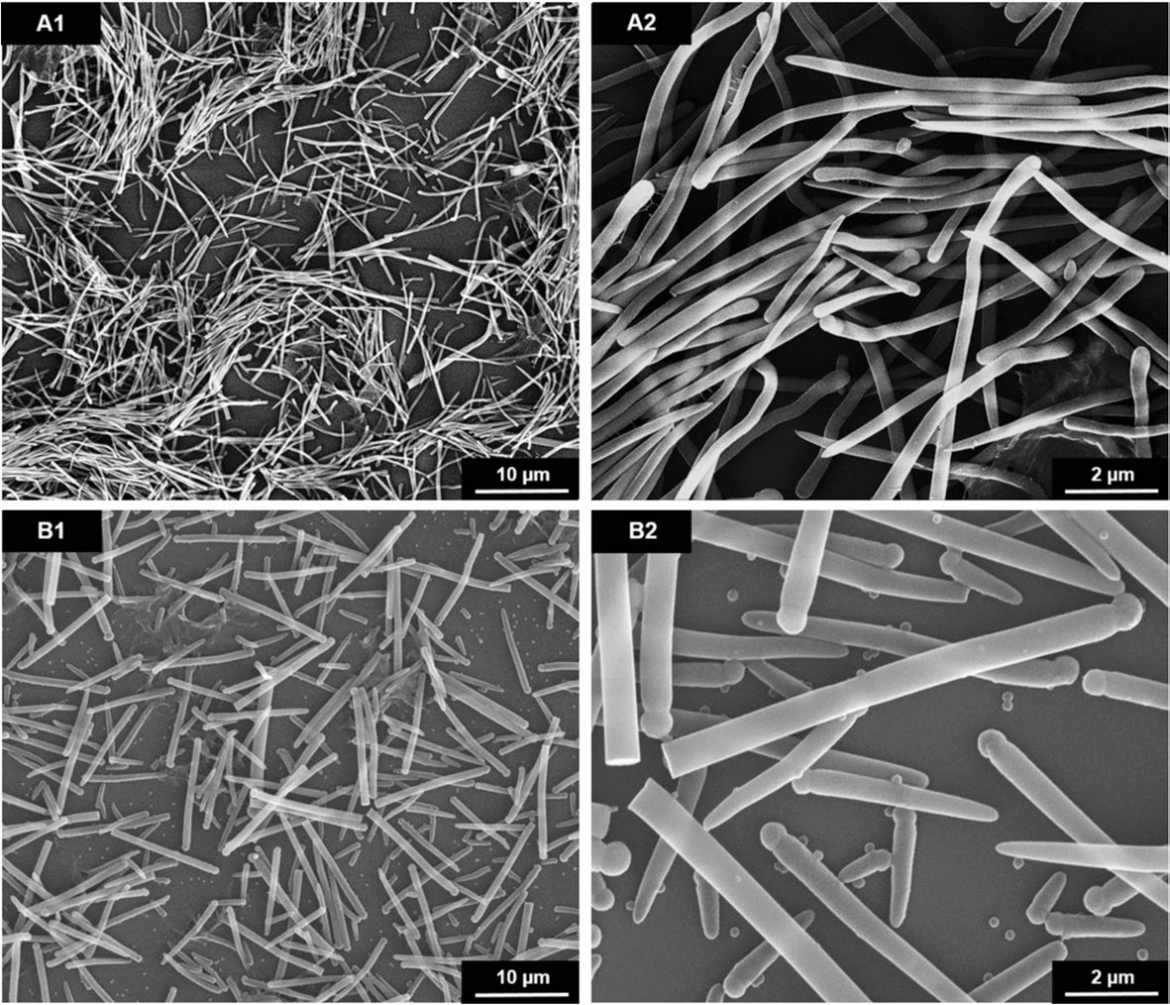
Representative SEM images at two different magnifications of straight thin (A1, A2) and straight thick (B1, B2) silica fibers, prepared by microemulsion synthesis

Synthetic silica fibers were prepared by an emulsion-based synthesis [17] which was adapted and varied from an earlier version [13] to obtain uniform and long fibers with high aspect ratio in high yield. Basically, it involved the nucleation of tetraethylorthosilicate (TEOS) on water droplets in a water-in-oil emulsion with 1-pentanol as oil phase and ethanol/water as dispersed water phase [13, 17]. The silica fibers grew into one direction after nucleation from the initial water droplet after hydrolysis of TEOS to silica. A systematic variation of the synthesis parameters showed that the ethanol-to-water ratio plays a key role in determining the shape of the silica fibers. Straight thick (∼6 µm ⋅ 0.35 µm; aspect ratio 17:1) and straight thin (∼8 µm ⋅ 0.25 µm; aspect ratio 32:1) silica fibers were prepared at ethanol-to-water ratios of 1.75:1 (*v*/*v*). In order to decrease the diameter of straight fibers, additional treatment of the reaction mixture by ultrasonication was carried out before TEOS addition to decrease the water droplet size for thin straight fiber nucleation. In some cases, the initial water droplet was still visible by SEM at the fiber end as a sphere after it had mineralized to silica (Fig. 1).

**Fig. 2:**
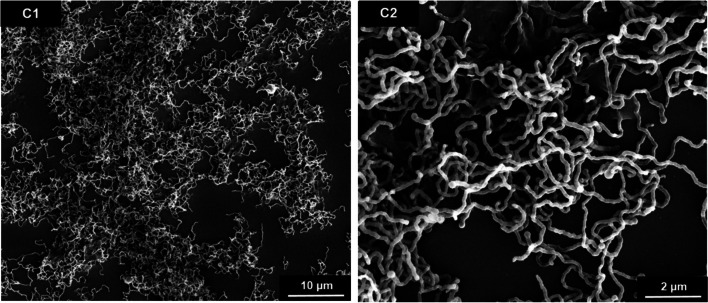
SEM images at two different magnifications of curly silica fibers prepared by microemulsion synthesis at a 3.5:1 (*v*/*v*) ethanol-to-water ratio

The increase of the ethanol-to-water ratio in the synthesis to 3.5:1 (*v*/*v*) led to curly silica fibers with a thickness of about 0.1 µm, i.e., generally thinner than the straight fibers (Fig. 2). The length was difficult to determine but usually around 9 µm or more, giving an aspect ratio of about 70:1. Length and width distributions of all silica fiber types are given in Supplementary Figure S1. The radius of curvature was about 0.5 µm as estimated from visual analysis of the SEM images (Fig. 2). However, as the curly fibers are diverse, this is just an approximate value. A further increase of the ethanol-to-water ratio beyond >3.5:1 resulted in ill-defined silica particles and silica microspheres (similar to the images shown in Supplementary Figure S3).

**Fig. 3:**
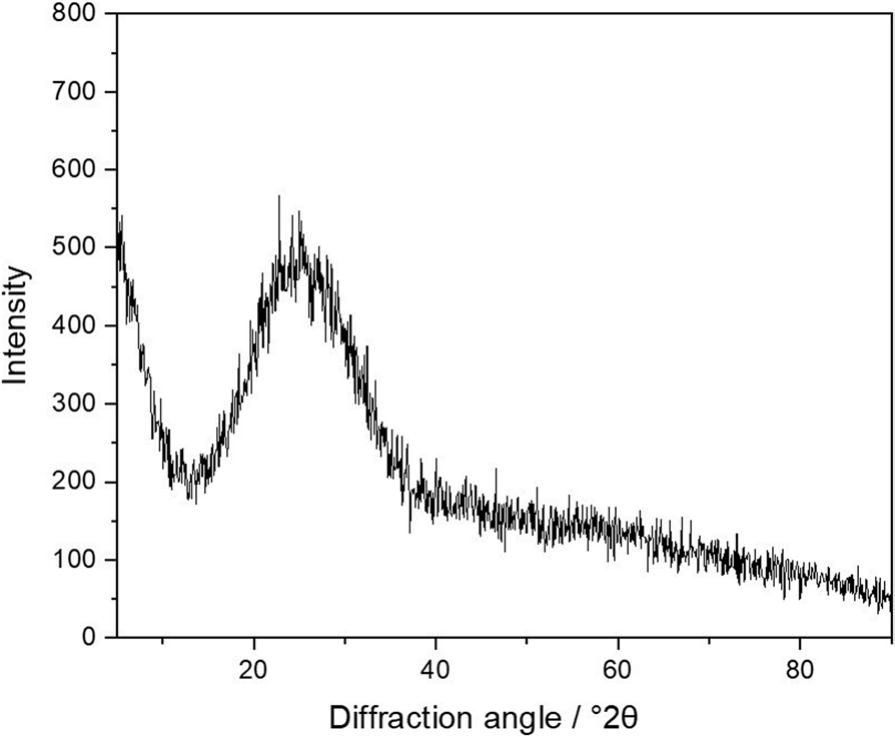
Representative X-ray powder diffractogram of straight thin silica fibers. The characteristic halo peak between 15-30 °2θ indicates the presence of amorphous silica. No peaks of crystalline phases were found. The powder diffractograms of all other fiber samples were very similar

All syntheses were well reproducible. The yield of a synthesis was between 10 and 40 mg for one batch. Upscaling the synthesis was not possible because shape and uniformity of the samples were lost (Supplementary Figure S3). All prepared fibers were fully X-ray amorphous as indicated by X-ray powder diffraction, i.e., they consisted of non-crystalline silica, SiO_2_·*x* H_2_O (Fig. 3).

### Release of silicate ions and long-term stability of the fibers upon immersion

The biological effects of silica fibers might be due to the release of silicic acid/silicate ions by slow dissolution which may be different for different particle types [23]. To elucidate this effect, solubility studies were performed to investigate the release of silicon (as silicate ions) from fibers immersed in DPBS. There was no obvious difference in the release rate of silicate ions between straight and curly silica fibers (Table 1). Between 4.7 and 5.8 wt% of the total silica was released after 7 days of immersion in DPBS. The silicate ion release determined for the spherical silica particles (used as reference particles in this study; size range of 10-20 nm) was close to the values of the silica fibers. The release rate of silicate ions from the quartz control sample that consisted of globular particles with an average grain size of 8 µm was about three times lower than from the silica fibers, obviously due to the lower solubility of this crystalline phase with its higher lattice energy [23]. As negative control, the pure incubation buffer (DPBS) was also examined for the presence of silicate ions (Table 1).

**Table 1:** Silicate ion release from silica fibers with different shape after immersion for 7 days at ambient temperature in DPBS at a fiber concentration of 1 mg mL^-1^ silica (1,000 ppm). Dissolved silicon can be present as silicate (SiO_4_^4-^), silicic acid (H_4_SiO_4_) or differently protonated silicate species.

Sample	Si concentration in the DPBS medium / ppm^a^	Dissolved fraction of sample / wt%	Dissolved fraction / wt% per day
Thin straight silica fibers	20.4 (46.9)	4.7	0.67
Thick straight silica fibers	22.2 (51.1)	5.1	0.73
Curly silica fibers	25.3 (58.2)	5.8	0.83
Silica nanoparticles	19.5 (44.9)	4.5	0.64
SH500 quartz	9.1 (19.5)	2.0	0.28
DPBS buffer (negative control)	2.5	-	-

^a^The values in brackets indicate the computed weight loss from the samples. This is based on the stoichiometric weight ratio of silicon to oxygen in amorphous silica, i.e., 1:2.3 for fibers and nanospheres as determined by EDX before, and 1:2.14 for quartz (silicon dioxide, SiO_2_; stoichiometric mass ratio). During the immersion, silicon was measured by ICP-MS.

**Fig. 4:**
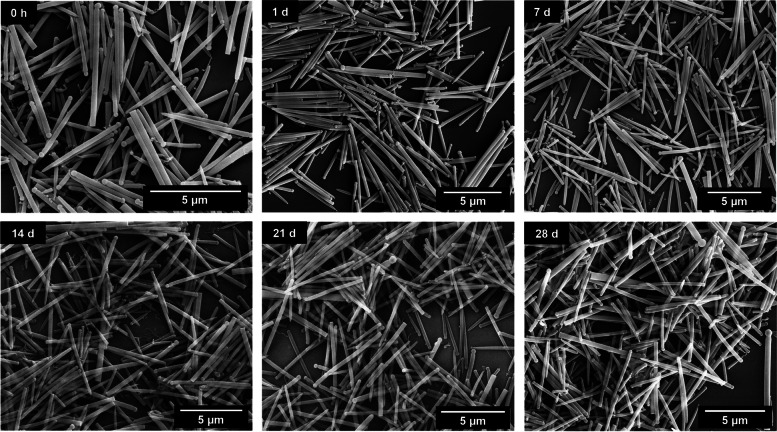
SEM images of straight thick silica fibers after immersion in water for up to 28 days

**Fig. 5:**
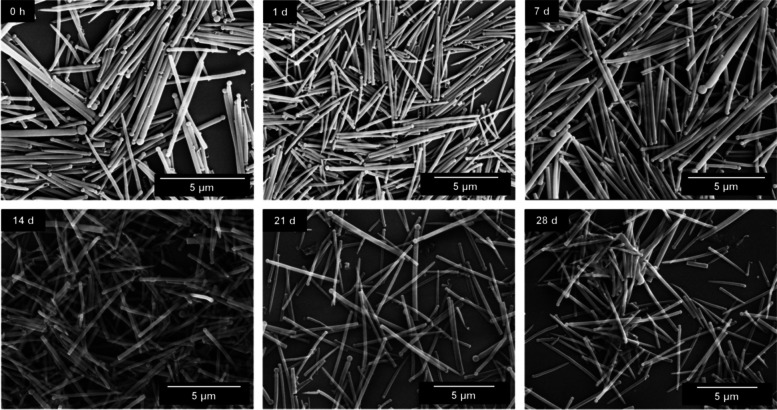
SEM images of straight thin silica fibers after dispersion in water for up to 28 days

**Fig. 6:**
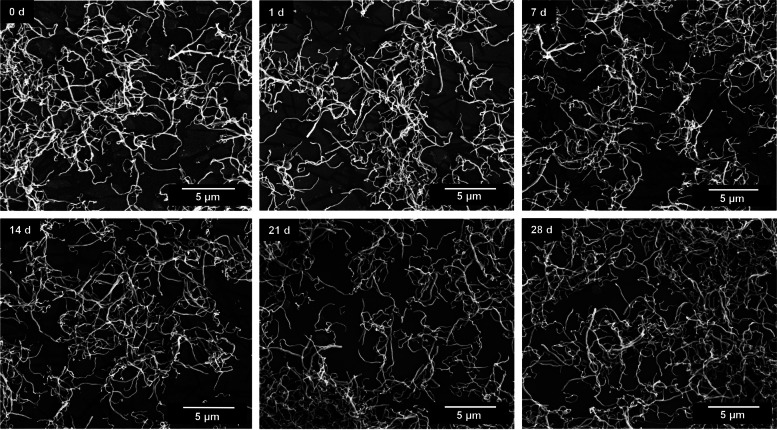
SEM images of curly silica fibers after dispersion in water for up to 28 days

Long-term immersion experiments in water showed that the silica fibers were stable upon immersion (Figs. 4, 5 and 6). Length and diameter distribution of the silica fibers after synthesis and after 28 days in immersion are given in the Supplementary Figure S2. No significant changes in the fiber shape were observed during 28 days of incubation, except for some broadening of the fiber length distribution, indicating a few broken fibers. This underscores that the amount of released silica was minor in comparison to the total fiber volume.

### Cell toxicity of silica fibers

**Fig. 7:**
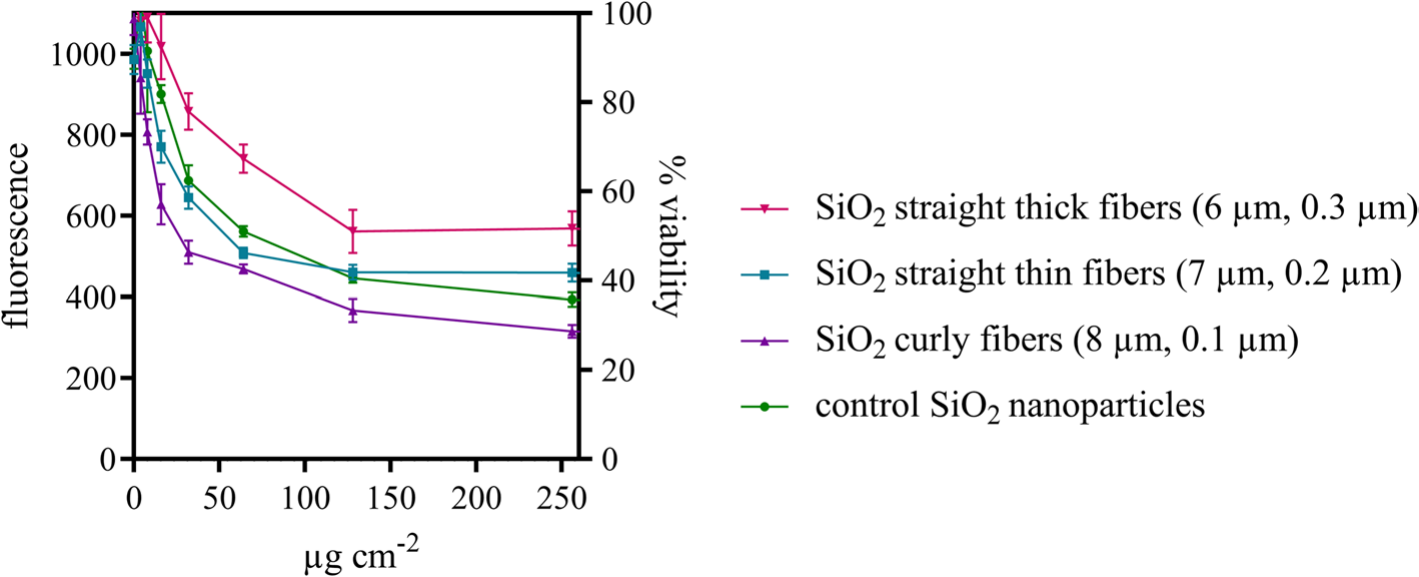
Cell viability (proportional to the fluorescence intensity) of NR8383 cells after incubation with silica fibers of different shape for 24 h as determined by the AlamarBlue^®^ assay, given against the administered dose of fibers. Three independent experiments were carried out for each sample (*N*=3). Silica nanoparticles were analyzed as granular particle control. The following IC50 values were calculated by non-linear regression analysis: 25 µg cm^-2^ (*R*^2^ = 0.814) for straight thin silica fibers, 14 µg cm^-2^ (*R*^2^ = 0.949) for curly silica fibers, and 49 µg cm^-2^ (*R*^2^ = 0.764) for straight thick silica fibers

Cell toxicity on NR8383 cells was determined for the three types of silica fibers with the AlamarBlue^®^ assay at concentrations up to 500 µg cm^-2^. Results are shown up to 250 µg cm^-2^. SiO_2_ nanoparticles were used as granular particle control. Straight thin fibers showed a higher cell toxicity than straight thick fibers. The comparatively strongest cell toxicity was observed for the curly fibers (Fig. 7). However, significant cytotoxicity was only observed at very high particle doses.

**Fig. 8:**
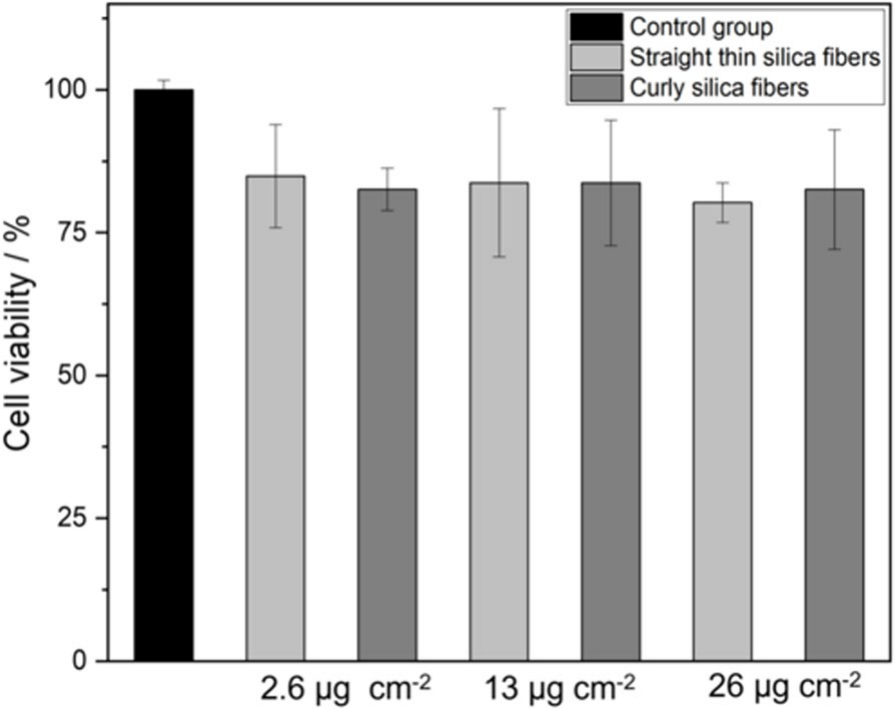
Viability assay (MTT test) after incubation of THP-1 cells with straight thin and curly silica fibers for 24 h, respectively

In good agreement, none of the fibers induced a strong cytotoxic effect on THP-1 cells at concentrations up to 26 µg cm^-2^ as shown by an MTT test (Fig. 8). The significance levels are given in Supplementary Table T2.

### Uptake of silica fibers by macrophages

**Fig. 9:**
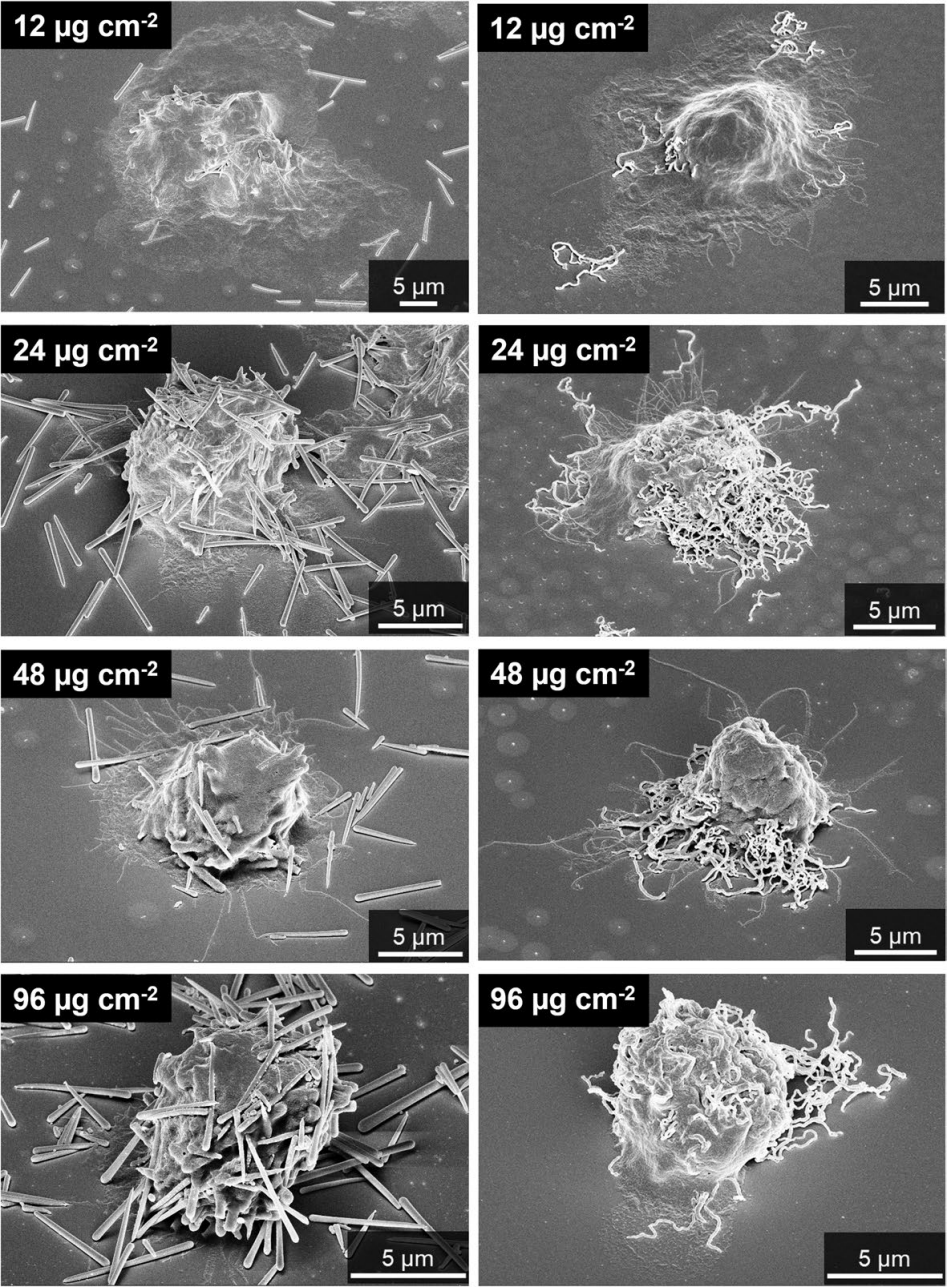
Uptake of straight thick (left) and of curly silica fibers (right) by NR8383 rat macrophages, incubated for 16 h with 12 µg cm^-2^, 24 µg cm^-2^, 48 µg cm^-2^, and 96 µg cm^-2^, visualized by scanning electron microscopy after thorough washing

The interaction of silica fibers with cells was assessed by scanning electron microscopy and confocal laser scanning microscopy. Besides a prominent sedimentation of the synthetized fibers onto the cells and the substrate, straight fibers as well as curly fibers were taken up by NR8383 rat macrophages (Fig. 9). Despite thorough washing after incubation and before SEM analysis, many fibers were still attached to the surface of the cells and the substrate.

**Fig. 10:**
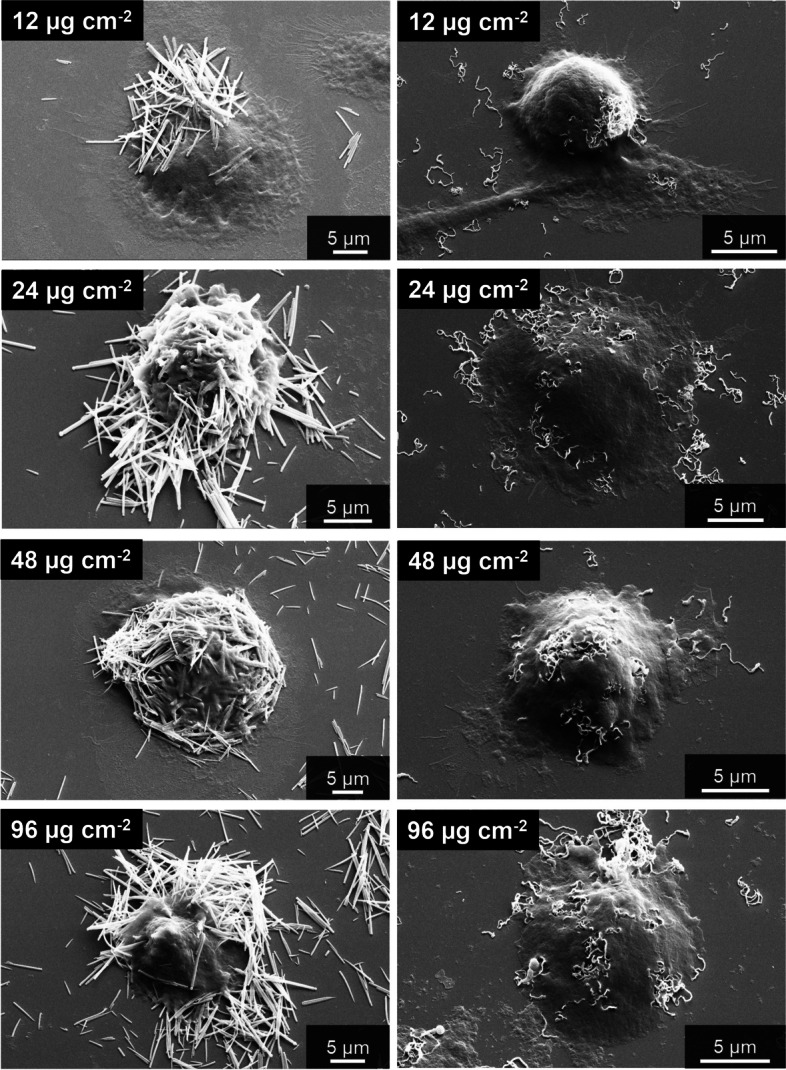
Uptake of straight thin (left) and curly silica fibers (right) by human THP-1 macrophages, incubated for 24 h with 12 µg cm^-2^, 24 µg cm^-2^, 48 µg cm^-2^, and 96 µg cm^-2^, visualized by scanning electron microscopy after thorough washing

Very similar results were observed for human THP-1 macrophages (Fig. 10). Straight and curly fibers were taken up by the cells. The fibers also sedimented onto the cells and the substrate. THP-1 macrophages were shown earlier to take up silica microfibers as well as barium sulphate microparticles [13, 24].

**Fig. 11:**
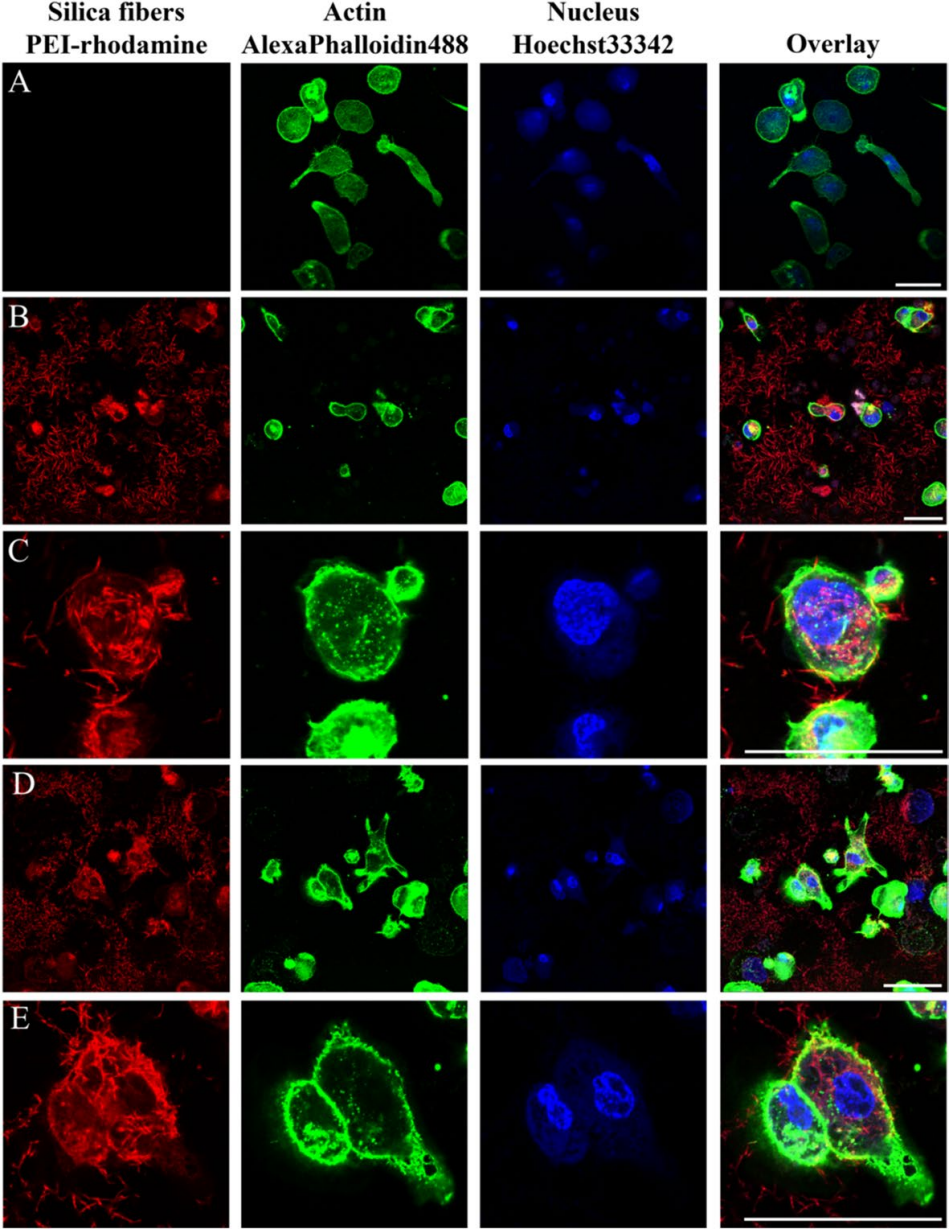
Uptake of straight thick and curly silica fibers by THP-1 cells by confocal laser scanning microscopy (24 h). Control group (without treatment with fibers) (**A**), 48 µg cm^-2^ straight thick fibers (**B**), close-up of straight thick fibers (3x zoom) (**C**), 48 µg cm^-2^ curly fibers (**D**), close-up of curly fibers (3x zoom) (**E**). Scale bars: 20 µm

Confocal laser scanning microscopy was used to visualize the uptake of fluorescently labelled fibers after phagocytotic uptake by human macrophages. Straight thick and curly fibers were present inside the THP-1 cells (Fig. 11).

The particles were localized within the cell by means of a 3D reconstruction of a confocal image of cells (see Supplementary Figure S6 for a representative example).

**Fig. 12:**
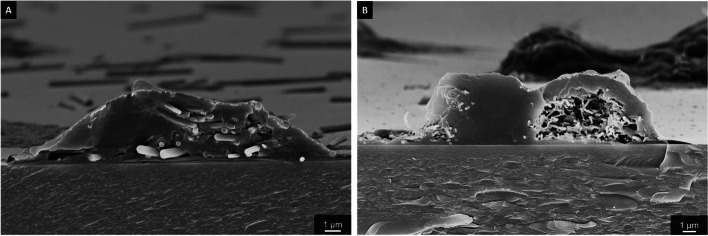
Representative SEM images of silica fibers inside dissected (freeze-fractured) NR8383 alveolar macrophages after incubation for 16 h at 12 µg cm^-2^: An NR8383 cell with straight thick fibers inside (A), an NR8383 cell with curly fibers inside (**B**)

**Fig. 13:**
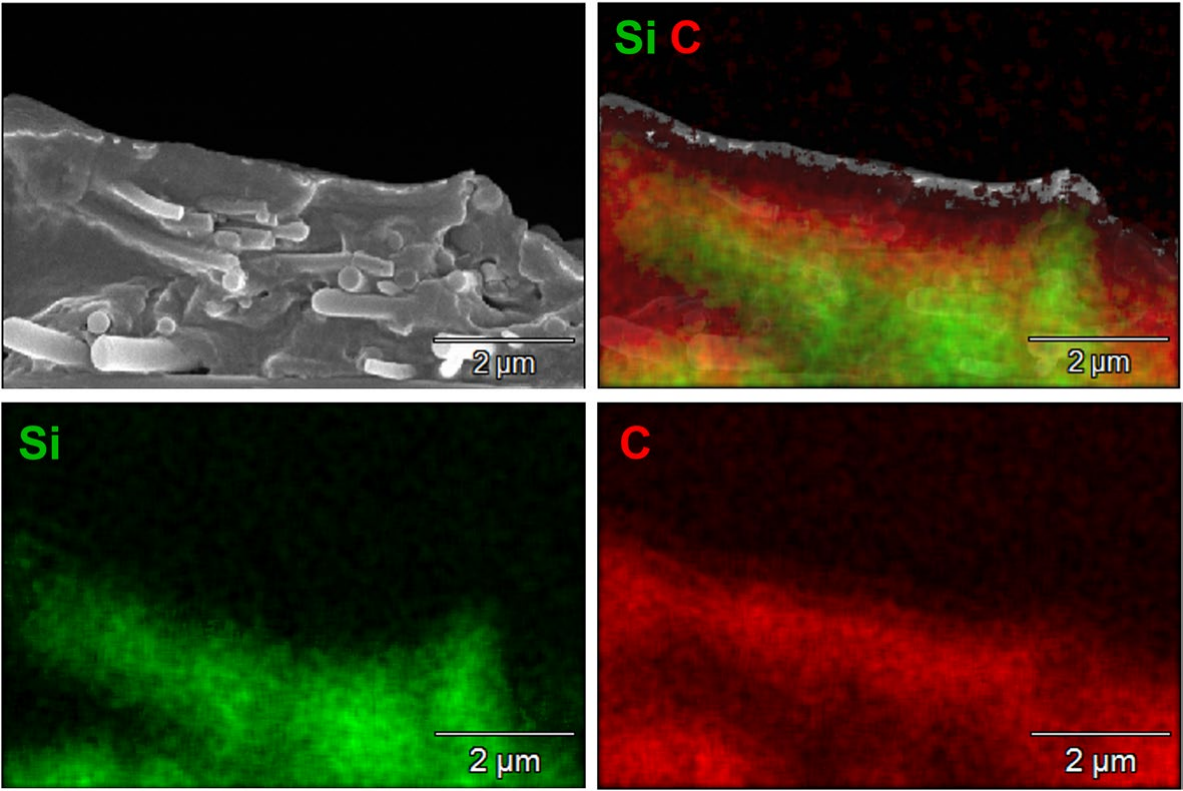
Representative EDX mapping of a dissected (freeze-fractured) NR8383 cell, incubated with straight thick fibers for 16 h at 12 µg cm^-2^. Silicon (fibers): green. Carbon (cell components): red

**Fig. 14:**
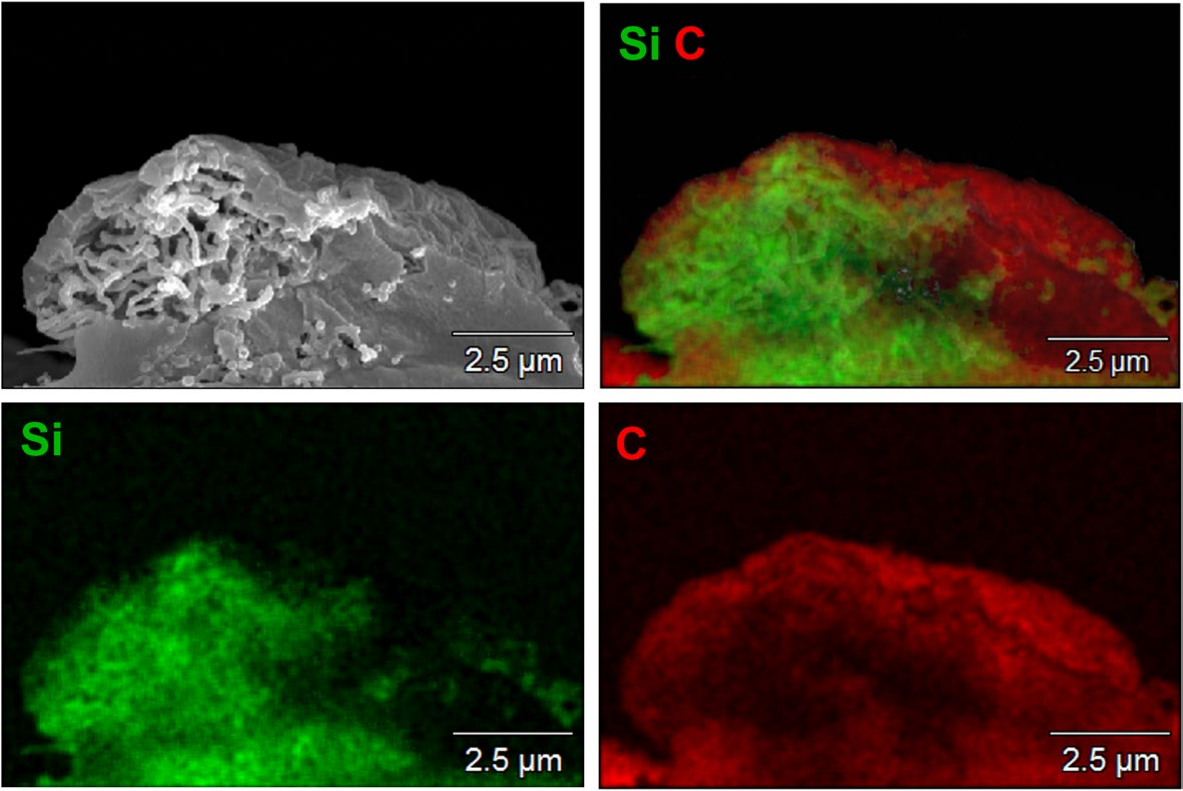
Representative EDX mapping of a dissected (freeze-fractured) NR8383 cell, incubated with curly fibers for 16 h at 12 µg cm^-2^. Silicon (fibers): green. Carbon (cell components): red

The efficient uptake of fibers was also shown by scanning electron microscopy in combination with EDX of NR8383 cells after freeze-fracturing that permitted a view into the cells (Figs. 12, 13 and 14).

**Fig. 15:**
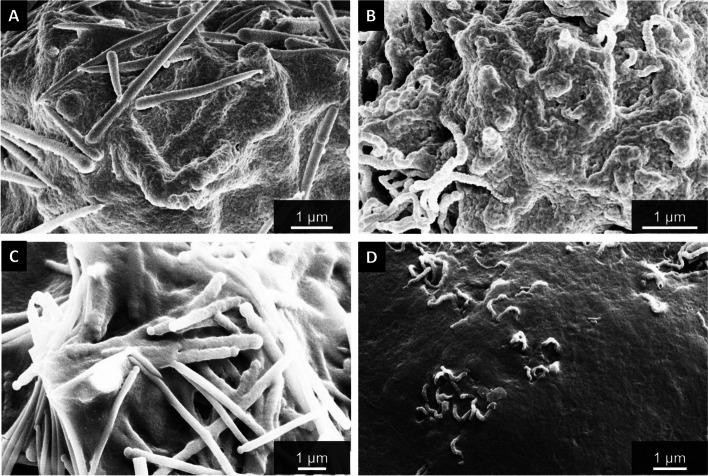
Representative magnifications of the interface between cell wall and fibers by SEM illustrate cell membrane damage after uptake: An NR8383 cell and straight thick fibers (A), an NR8383 cell and curly fibers (**B**), both incubated for 16 h with 48 µg cm^-2^; a THP-1 cell and straight thin fibers (**C**), and a THP-1 cell and curly fibers (**D**), both incubated for 24 h with 48 µg cm^-2^

The cell membrane of both types of macrophages was clearly damaged by the fibers as shown by higher resolved scanning electron microscopy (Fig. 15). Some fibers were not completely engulfed by the macrophages and partially penetrated the cell wall.

When it comes to the contact of the fibers with adherent cells, the sedimentation rate in the cell culture well is of prime importance [25]. Larger particles are predominantly transported via gravitational settling [26]. The micrometer size of the fibers leads to rapid sedimentation. To estimate the sedimentation time of a silica microfiber in water, the gravitationally driven sedimentation rate was computed by Stokes' law

$$v=\frac{\mathrm{g }\left({\rho }_{p}-{\rho }_{f}\right) {d}^{2}}{18 \mu }$$

with *v* the sedimentation rate (m s^-1^), g the standard earth gravity (9.81 m s^-2^), ρ_*p*_ the particle density (assumed as 2,650 kg m^-3^, i.e. the density of quartz), ρ_*f*_ the fluid density (assumed as 1,000 kg m^-3^, i.e. the density of water), *d* the particle diameter (here a typical fiber length of 4 µm = 4·10^-6^ m was tentatively used, given the fact that Stokes' law assumed spheres), and μ the dynamic viscosity of the medium (assumed as 1 mPa s, i.e. the viscosity of water). The estimated time needed for a fiber to sediment from the top of the cell culture dish to the bottom (*h* assumed with 3 mm = 3·10^-3^ m) with the computed sedimentation rate (*v* = 1.4·10^-5^ m s^-1^) is approximately 210 s. This means that all fibers have settled to the bottom of the well within the incubation time of 16 h, and that the adherent cells are covered by fibers already at the start of the cell culture experiment. This also corresponds to visual inspection of the samples, i.e. the approximation of spherical particles can be tentatively transposed to fibers. We also computed the theoretical dose of fibers per cell, the fiber surface area per cell, and the fiber volume per cell (Table 2). The number of fibers per cell is considerable, as it is also evident from the microscopic images, even after washing the cells and removing most fibers. A simple geometric assessment shows that 19, 29, and 58% of the bottom area of the well would be covered, by straight thick, straight thin, or curly fibers, respectively, if the fibers formed a close aligned packing. As they will form an irregular heap, as in the SEM images, it is clear that the actual local dose of fibers is very high. With respect to the fiber uptake by macrophages as investigated by SEM and confocal microscopy (Figs. 9, 10, 11, 12, 13, 14 and 15), it is not possible to give the actual number of fibers in each cell. However, as the cells were washed after incubation and before microscopy, the number of particles taken up is definitely lower than the maximum dose given in Table 2.

**Table 2:** Dose of silica fibers per well and per cell, assuming a cylindrical particle shape. The calculation was performed for a dose of 12 µg cm^-2^ of fibers and a cell density of 2.4∙10^5^ per cm^-2^. Due to sedimentation, all cells are rapidly covered within minutes by sedimenting fibers

Silica fibers	Dimension of one fiber / µm	Volume of one fiber / µm^3^	Mass of one fiber / kg	Surface area of one fiber / µm^2^	Number of fibers per cell	Surface area of fibers per cell / µm^2^	Volume of fibers per cell / µm^3^
Straight thick	6·0.3	0.42	1.12·10^-15^	5.66	44	252	19
Straight thin	7·0.2	0.22	5.83·10^-16^	4.40	86	377	19
Curly	8·0.1	0.063	1.66·10^-16^	2.51	300	755	19

## Chemotaxis

**Fig. 16:**
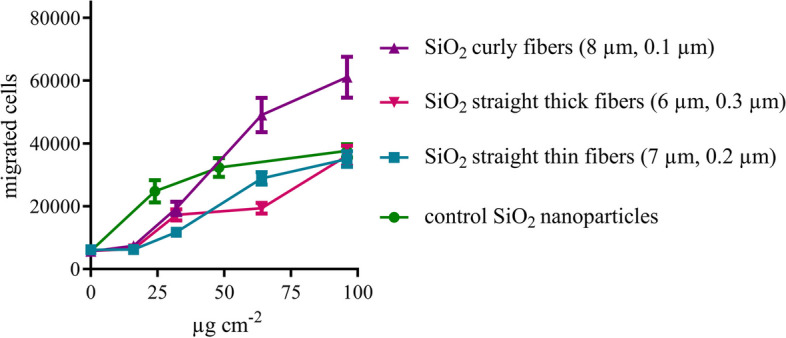
Induction of chemotaxis by silica fibers of different length, thickness, and shape as assessed by PICMA. The migration of dHL-60 cells (migrated cells) induced by cell supernatants of nanoparticle-incubated macrophages is plotted against the dose of fibers (in µg cm^-2^). Averages of three independent experiments are shown (*N*=3). Silica nanoparticles served as a control

PICMA served as an estimate of inflammatory reactions at sub-toxic concentrations. Inflammatory responses are characterized by the accumulations of inflammatory cells (especially neutrophilic granulocytes) that are attracted to the site of inflammation by stressed macrophages (Fig. 16). Further data of individual particle batches are given in Figure S3, illustrating the high reproducibility of the observed effects.

Curly fibers clearly induced the strongest migration of dHL-60 cells of all prepared fibers. Straight thick and thin fibers showed lower chemotaxis, comparable to control SiO_2_ nanoparticles. Significantly different induction of chemotaxis was observed for curly and straight thin SiO_2_ fibers.

## RNA expression analysis

For the expression analyses of inflammatory mediators, RNA was isolated from the rat macrophages after exposure to straight thick fibers or curly fibers. For normalization of raw data, a combination of *B2m* and *Ldha* was identified as the most valuable reference. Expression analysis of 84 chemokines and cytokines revealed 27 genes that showed a statistically significant changed expression after exposure of NR8383 cells to straight thick fibers or curly fibers (Table 3).

**Table 3:** Statistically significant up- (green) and downregulated (red) genes after exposure of NR8383 cells to straight thick fibers and curly fibers for 16 h (FC: fold change).

**Gene**	**Straight fibers (16 µg cm**^**-2**^**)** **FC and *p*-value**		**Straight fibers (32 µg cm**^**-2**^**)** **FC and *p*-value**		**Curly fibers (16 µg cm**^**-2**^**)** **FC and *p*-value**		**Curly fibers (32 µg cm**^**-2**^**)** **FC and *p*-value**	
*Ccl12*	0.38	0.001	0.51	0.005	1.12	0.278	2.47	0.004
*Ccl17*	1.51	0.005	1.27	0.052	1.65	0.101	2.13	0.008
*Ccl2*	2.25	0.243	105.90	<0.001	233.81	<0.001	363.54	<0.001
*Ccl22*	2.86	0.005	4.00	0.005	2.88	0.004	4.37	0.005
*Ccl24*	0.46	0.086	0.57	0.082	1.33	0.259	5.01	0.001
*Ccl3*	5.41	<0.001	16.48	<0.001	23.75	0.008	54.71	<0.001
*Ccl4*	19.19	<0.001	65.24	<0.001	130.90	<0.001	251.80	<0.001
*Ccl5*	1.54	0.161	3.35	0.022	2.51	0.136	4.36	0.021
*Ccl7*	1.84	0.085	4.91	0.012	10.21	0.050	14.84	0.013
*Cd40lg*	0.36	0.025	0.28	0.011	0.35	0.016	0.35	0.013
*Csf1*	1.35	0.092	2.47	0.034	2.62	0.074	6.88	0.004
*Ctf1*	0.54	0.061	0.43	0.026	0.43	0.033	0.39	0.018
*Cxcl1*	33.61	0.012	125.90	0.005	385.25	<0.001	820.07	0.003
*Cxcl10*	0.61	0.026	0.45	0.008	0.73	0.178	0.68	0.03
*Gdf15*	26.18	0.007	97.98	0.002	153.52	0.004	311.46	<0.001
*Il15*	0.62	0.003	0.36	<0.001	0.43	<0.001	0.18	<0.001
*Il17f*	13.15	0.013	23.38	0.024	36.49	0.011	121.38	0.020
*Il1a*	2.91	0.006	5.36	<0.001	9.42	<0.001	18.75	0.002
*Il1b*	2.18	0.001	2.72	0.006	5.39	<0.001	8.30	0.001
*Il1rn*	2.46	0.007	4.21	<0.001	4.11	0.008	7.13	0.018
*Il23a*	4.44	<0.001	13.95	0.005	41.65	0.001	105.32	<0.001
*Il6*	1.18	0.591	5.10	0.023	5.86	<0.001	9.23	0.034
*Il7*	0.94	0.563	0.59	0.011	0.52	0.017	0.27	<0.001
LOC103694381	0.54	0.096	0.34	0.003	0.43	0.019	0.23	0.002
*Tgfb2*	2.48	0.002	4.23	0.006	3.17	0.009	4.80	0.013
LOC103694380^a^	10.86	0.003	41.10	0.001	91.41	<0.001	184.26	<0.001
*Tnfsf10*	0.42	0.061	0.08	0.010	0.11	0.013	0.07	0.009

^a^*Tnfα*

**Fig. 17:**
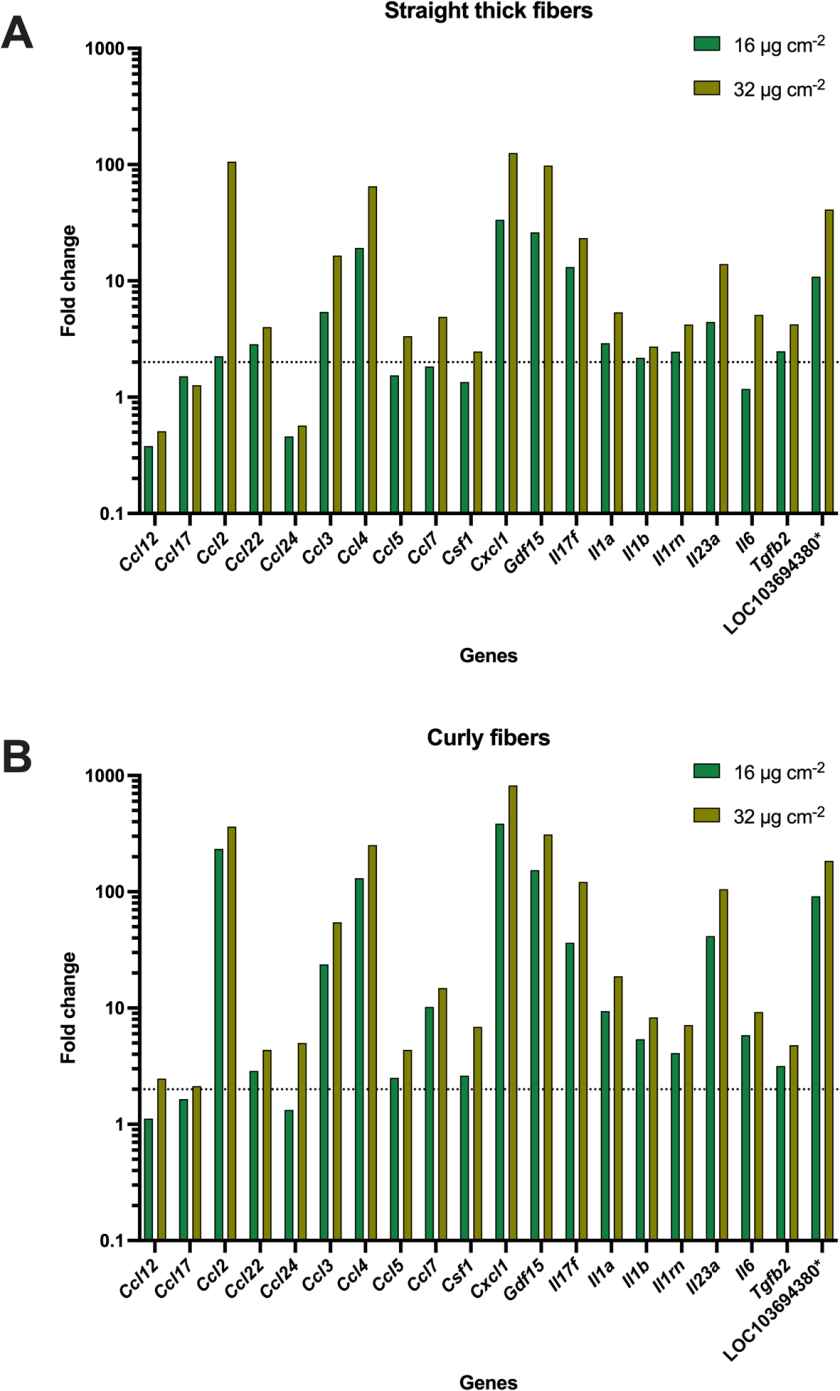
Dose-dependent fold changes of the induced genes after exposure of NR8383 cells to straight thick silica fibers (**A**) and curly silica fibers (**B**). The fold change is represented in log scale. Dotted lines represent the statistically significant cut-off of FC>2.0 for upregulation (*LOC103694380 is also known as *Tnfα*)

A downregulation was observed for six genes, depending on the fiber morphology as well as on the concentration, whereas for most genes (*N*=19), an upregulation, i.e., an induction, was observed, e.g., for *Gdf15*, *Tgfb2*, and LOC103694380 (known as *Tnfα*). Overall, the FC was increased in a dose-dependent manner after exposure to straight thick fibers (Fig. 17A) as well as curly fibers (Fig. 17B). Additionally, in comparison of the two fibers morphologies, the FC was generally higher after exposure to curly fibers in contrast to straight thick fibers.

## Discussion

The WHO fiber definition includes fibers of different toxicity [1–3]. Unfortunately, the parameters that determine fiber toxicity are not sufficiently known: "The presently available data do not make it possible to state precisely from which fiber length or diameter or from which length to diameter ratio and from which durability fibers possess the biological activity resulting in tumor induction" [27]. Frustrated phagocytosis is surely a key patho-mechanistic event of fiber toxicity. The opinions from which length this mechanism comes into effect vary in the literature, and up to 20 µm length were suggested. Some authors even doubted that short mineral fibers contribute to carcinogenic effects at all [9, 28]. According to a meta-analysis, the highest risk of lung cancer was observed for amphiboles >10 µm in length. Longer and thinner fibers were also more pathogenic for mesothelioma, especially exposure towards amphiboles ≥5 µm [29]. Other authors reported that mineral dust with a fibrous component is primarily carcinogenic if it contains amphibole fibers longer than 5 µm [4] and thinner than 0.25 µm [30] For health protection purposes it would therefore be helpful to know the exact preconditions for inflammatory, carcinogenic, and fibrinogenic effects from fiber exposure. The investigation of the parameters that may contribute to fiber toxicity appears to be best achieved via comparative *in vitro* studies. *In vivo* studies are not always possible because the targeted synthesis of fibers often does not produce sufficient quantities, i.e. the test materials are available only to a limited extent. An advantage of doing comparisons with synthetic fibers is the discrete size distribution that can be achieved, whereas naturally occurring fibers often show a continuous spectrum of different diameters, lengths, and possibly chemical composition.

Therefore, well-defined synthetic silica fibers were prepared here. Earlier, we had reported the synthesis and biological effects of short silica microfibers (3.2 µm·0.3 µm) with an aspect ratio of about 10:1 [13]. The synthesis of silica fibers with different aspect ratio and curvature was possible by adaption of a microemulsion synthesis with high reproducibility. The aspect ratio was increased to 17:1 and 32:1 for two samples of straight fibers and to 70:1 for curly fibers with fiber diameters down to 0.25-0.35 µm and 0.13 µm, respectively. However, we found that the synthesis was very sensitive to the reaction conditions, e.g., vessel size, rate of reagent addition and dilution, and diffusion/convection effects. This is not surprising, given the slow growth of the silica fibers into the emulsion which must not be disturbed, e.g., by convection. Attempts for upscaling were not successful, therefore it was necessary to perform parallel syntheses and then to pool the samples after thorough characterization to obtain sufficient material for the following analyses. The typical yield of 10 to 40 mg per synthesis corresponds to about 10^10^ fibers per batch. It remains open why the fibers are reproducibly curved under some specific synthesis conditions. As they were all X-ray amorphous, they were formed by a sol-gel-type hydrolysis of TEOS to amorphous silica. An influence of the crystal structure as it is the case with curved chrysotile asbestos fibers [10] can be ruled out due to the isotropic nature of the amorphous fiber mineral (silica). Nevertheless, the fibers are well suited for model studies on the effect of microfibers on cells.

The cell toxicity of the synthesized silicate fibers corresponded approximately to that of granular biopersistent particles such as silica nanoparticles as reported earlier [15, 31, 32]. The computed particle surface area per cell was between 250 and 750 µm^2^, i.e., well below the limit given by Wiemann et al. for a non-toxic interaction [33]. However, the fibers sedimented within minutes onto the cells, forming a layer on the cells, therefore we can assume that any negative effect is caused by an overload of cells by fibers which is also apparent on the SEM images of cells after incubation with fibers. Intrinsic cytotoxic effects are unlikely, also given the chemical nature of the fibers that consist exclusively of non-toxic silica [32, 34, 35] with a small amount of non-toxic PVP [36].

As some reports are pointing to a contribution of residual solubility to particle toxicity, including granular silica [23, 37–39], we measured the solubility of the fibers. The release of silica was very small and comparable for all fibers, therefore the solubility most probably did not contribute to the biological effects. The release of silicon from the fibers was about three times higher compared to crystalline quartz, as expected due to the amorphous nature of hydrated silica [23]. Additional insight could be gathered by performing perfusion experiments in biomimetic environments to assess the solubility under dynamic conditions as described in ref. [40]. For silica nanoparticles of different size and shape, we have reported earlier only a moderate induction of reactive oxygen species (ROS) formation [32]. Thus, an induction of fiber cytotoxicity by ROS formation as observed with iron-containing asbestos fibers [10] can be excluded. Consequently, the model silica fibers are acting only due to their shape and size and not by an intrinsic chemical or cytotoxic activity.

We comprehensively assessed the biological effects by monitoring the fiber uptake, the particle-induced cell migration as an *in vitro* measure for inflammatory effects, and the expression of chemokines and cytokines to compare structural fiber parameters that may influence fiber toxicity. All fibers were taken up by NR8383 and THP-1 macrophages to a high extent with no significant differences between the fiber types. Due to their size and density, the fibers settled within minutes onto the adherent macrophages, clearly causing a very high local fiber concentration that induced phagocytosis [41, 42]. The observed cell membrane damage by the long fibers probably contributed to the observed cytotoxicity, but clearly did not cause strong adverse results, except at very high doses. Notably, the fibers remained intact under the conditions of cell culture, i.e., there was no significant breakup or other deterioration seen by SEM or CLSM.

The synthesized silica fibers caused only moderate chemotaxis with an extent that was of the same order as that of granular particles like silica nanoparticles [15, 32] but markedly weaker compared to asbestos fibers or MWCNT [16, 43]. In fact, the strongly carcinogenic MWCNT are very thin with diameters well below 100 nm, although some of them are relatively short. The straight fibers were slightly rounded at the ends due to the synthesis process. This may contribute to these fibers being less effective compared to MWCNT or asbestos fibers [16]. The chemotaxis was weaker than the effect of zinc oxide nanoparticles of different size and shape that was attributed to the release of zinc ions [44]. In contrast, titania nanoparticles had a smaller effect than the silica fibers, probably due to their chemical stability and the absence of dissolution [45], in agreement with fully inert barium sulphate particles from the nano- to the microscale [24, 46]. The chemotaxis of the synthetic fibers considered here was higher than with thicker silica microfibers of lower aspect ratio (length 3.2 µm; diameter 0.3 µm; aspect ratio 10:1) [13], suggesting an effect of the fiber dimension, including the aspect ratio.

The induction of chemotaxis was accompanied by the expression of numerous chemokines and cytokines as inflammatory mediators. A functionality of these mediators is likely because the fibers only slightly reduced viability, except at very high doses. Thus, it is unlikely that these genes were expressed as a result of cell toxicity. Furthermore, the dose-response of chemotaxis increased for most of these inflammatory mediators. The curly fibers had the strongest effects on these parameters. TGF-β (Tgfb2) was strongly induced. TGF-β is a chemokine that plays a crucial role in the pathogenesis of fibrosis [47, 48]. It is remarkable that TGF-β becomes visible in an *in-vitro* cell culture experiment. Growth differentiation factor-15 (GDF-15, Gdf15) showed one of strongest induction of all investigated signaling mediators. Functionally (not evolutionary), GDF-15 can be assigned to TGF-β. GDF-15 is expressed in numerous immunological contexts, such as infections, inflammation, and age-associated disorders of immune functions [49] as well as idiopathic fibrosis [50]. Overall, a part of the mediators which increased together with chemotaxis are associated with diseases that are also promoted by particle exposure, in particular lung fibrosis. This cell culture model thus is well suited to investigate early particle effects. The consistency of the results on cell toxicity, chemotaxis, and gene expression shows that the observed differences of effects between the straight and curly fibers are not random. It is not clear which of the inflammatory mediators directly influenced chemotaxis, but it appears that there is a cooperative effect.

Curly fibers were more cytotoxic, caused pronounced stronger chemotaxis, and accordingly stronger induction of the expression of inflammatory genes in NR8383 cells. Curly fibers were also the thinnest and longest fibers in these experiments, but it remains unclear whether the curvy shape or the aspect ratio caused these effects. It is also likely that the curly fibers are more easily bent than the straight fibers which may change the fiber effect on cells. Apparently, the incomplete uptake of the fibers leads to leaky cell membranes. This can be interpreted as an alternative mechanism of frustrated phagocytosis.

## Conclusions

We established a robust synthetic protocol to prepare silica fibers with different shape and aspect ratio. Differences of cytotoxicity, chemotaxis, and gene expression indicate an influence of fiber dimension on exposed macrophages. Although we formally synthesized WHO fibers, we did not reach a fiber length, diameter, or aspect ratio that strongly enhanced fiber toxicity, at least not within the time scale of our investigations (24 h). It is possible that there is a threshold from which the inflammatory potential of fibers rises sharply, above the length or below the diameter of the synthesized fibers, or both. In addition, the shape of the fibers may influence their toxic and inflammatory properties as indicated by curly fibers being significantly more active than straight fibers.

### Supplementary Information

Supplementary Material 1. RNA concentration, absorbance, and RNA integrity number (RIN) of the isolated NR8383 cells after incubation with silica fibers (Table S1), statistical analysis of MTT test with human macrophages (Table S2), histograms of fiber length and fiber width distribution as-prepared (Figure S1) and after 28 d immersion in water (Figure S2), results of unsuccessful upscaling syntheses (Figure S3), reproducibility of PICMA measurements (Figure S4), reproducibility of the Alamar Blue assay (Figure S5), 3D reconstruction of confocal laser scanning microscopy of THP-1 cells incubated with silica fibers (Figure S6).

## Data Availability

No datasets were generated or analysed during the current study.
