# Supplementary material for: Synthetic silica fibers of different length, diameter and shape: synthesis and interaction with rat (NR8383) and human (THP-1) macrophages in vitro, including chemotaxis and gene expression profile
Source: Part Fibre Toxicol. 2024 May 11;21:23. doi: 10.1186/s12989-024-00586-6 (PMC11088073; doi:10.1186/s12989-024-00586-6)
Supplement: Supplementary file 1 — Supplementary Material 1. RNA concentration, absorbance, and RNA integrity number (RIN) of the isolated NR8383 cells after incubation with silica fibers (Table S1), statistical analysis of MTT test with human macrophages (Table S2), histograms of fiber length and fiber width distribution as-prepared (Figure S1) and after 28 d immersion in water (Figure S2), results of unsuccessful upscaling syntheses (Figure S3), reproducibility of PICMA measurements (Figure S4), reproducibility of the Alamar Blue assay (Figure S5), 3D reconstruction of confocal laser scanning microscopy of THP-1 cells incubated with silica fibers (Figure S6). [file 12989_2024_586_MOESM1_ESM.pdf]

# Supplementary Information

**Synthetic silica fibers of different length, diameter and shape: Synthesis and interaction with rat (NR8383) and human (THP-1) macrophages in vitro, including chemotaxis and gene expression profile**

Nataniel Białas,[1,+] Nina Rosenkranz,[2,+] Daniel Gilbert Weber,[2,+] Kathrin Kostka,[1] Georg Johnen,[2] Aileen Winter,[1] Alexander Brik,[2] Kateryna Loza,[1] Katja Szafranski,[2] Thomas Brüning,[2] Jürgen Bünger,[2] Götz Westphal[2,*] and Matthias Epple[1,*]

[1] Inorganic Chemistry and Center for Nanointegration Duisburg-Essen (CENIDE), University of Duisburg-Essen, 45117 Essen, Germany

[2] Institute for Prevention and Occupational Medicine of the German Social Accident Insurance, Institute of the Ruhr University Bochum (IPA), 44789 Bochum, Germany

[+] Nataniel Białas, Nina Rosenkranz, and Daniel Gilbert Weber share first authorship.

[*] Correspondence authors: Matthias Epple, matthias.epple@uni-due.de, and Götz Westphal, goetz.westphal@dguv.de

**Table S1.** RNA concentration, absorbance, and RNA integrity number (RIN) of the isolated NR8383 cells after incubation with silica fibers.

| **Sample** | **Exposure** | **Replication** | **ng µL$^{-1}$** | **A260/A280** | **A260/A230** | **RIN** |
|---|---|---|---|---|---|---|
| Control | - | 1 | 2088 | 2.11 | 1.96 | 9.8 |
| | | 2 | 1793 | 2.11 | 2.25 | 9.7 |
| | | 3 | 1060 | 2.12 | 2.23 | 9.7 |
| Straight thick | 16 µg cm$^{-2}$ | 1 | 591 | 2.12 | 2.05 | 9.4 |
| silica fibers | | 2 | 593 | 2.11 | 2.23 | 9.8 |
| | | 3 | 592 | 2.13 | 1.96 | 9.7 |
| | 32 µg cm$^{-2}$ | 1 | 371 | 2.08 | 1.54 | 9.7 |
| | | 2 | 499 | 2.11 | 2.24 | 9.7 |
| | | 3 | 323 | 2.10 | 0.87 | 10 |
| Curly | 16 µg cm$^{-2}$ | 1 | 360 | 2.12 | 1.69 | 9.6 |
| silica fibers | | 2 | 440 | 2.08 | 2.26 | 9.9 |
| | | 3 | 349 | 2.11 | 1.36 | 9.6 |
| | 32 µg cm$^{-2}$ | 1 | 316 | 2.10 | 2.17 | 9.9 |
| | | 2 | 258 | 2.12 | 2.05 | 9.5 |
| | | 3 | 235 | 2.12 | 1.95 | 9.3 |

**Table S2.** Statistical *t*-test of the MTT assay after incubation of THP-1 human macrophages with silica fibers, compared to the control (untreated cells).

| **Fibre-type** | **Concentration / µg cm$^{-2}$** | ***p* (one-sided)** | ***p* (two-sided)** |
|---|---|---|---|
| Straight thin | 2.6 | 0.10 | 0.20 |
| Straight thin | 13 | 0.16 | 0.31 |
| Straight thin | 26 | 0.04 | 0.07 |
| Curly | 2.6 | 0.02 | 0.04 |
| Curly | 13 | 0.12 | 0.24 |
| Curly | 26 | 0.10 | 0.21 |

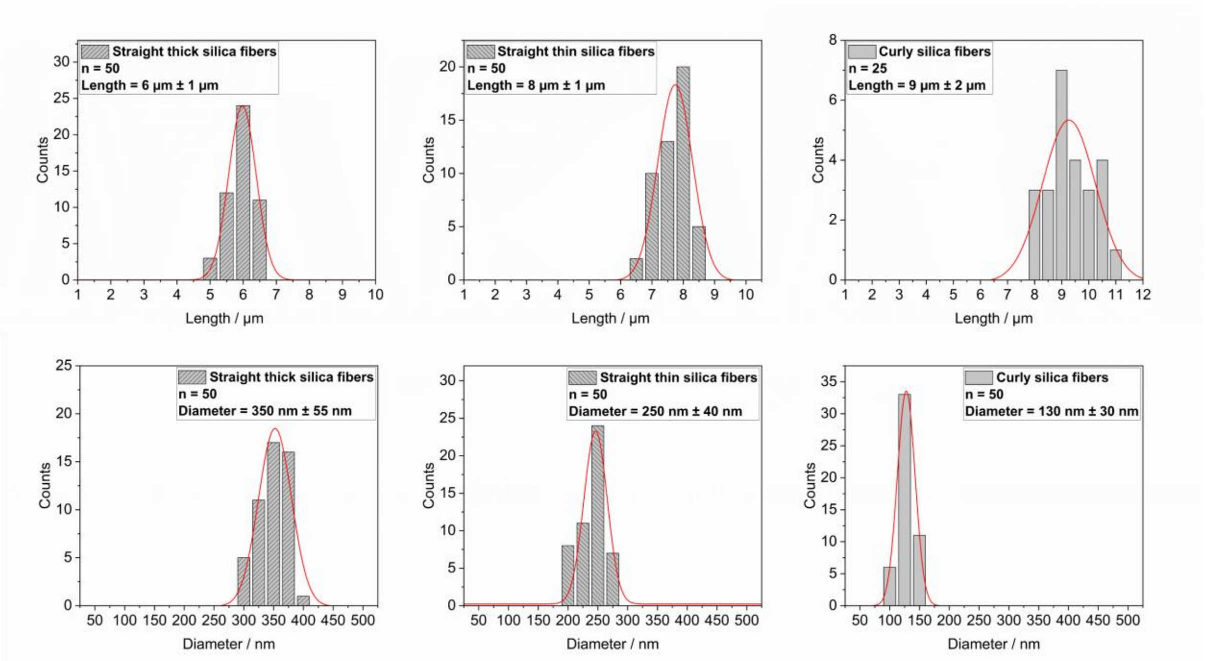


**Figure S1:** Histograms representing length (upper row) and width (lower row) distribution of as-prepared straight thick (left), straight thin (center) and curly (right) silica fibers. The average values and standard deviations were determined by Gaussian fits.

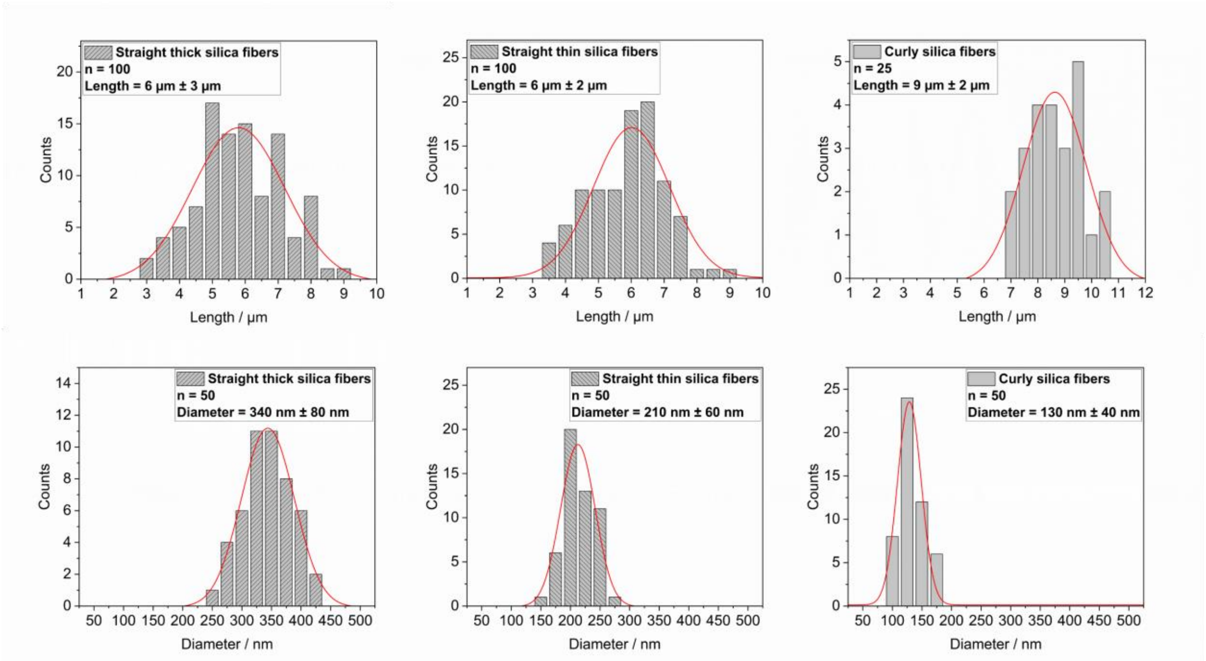


**Figure S2**: Histograms representing length (upper row) and diameter (lower row) distribution of straight thick (left), straight thin (center) and curly (right) silica fibers after 28 days in water immersion at ambient temperature. The average values and standard deviations were determined by Gaussian fits.

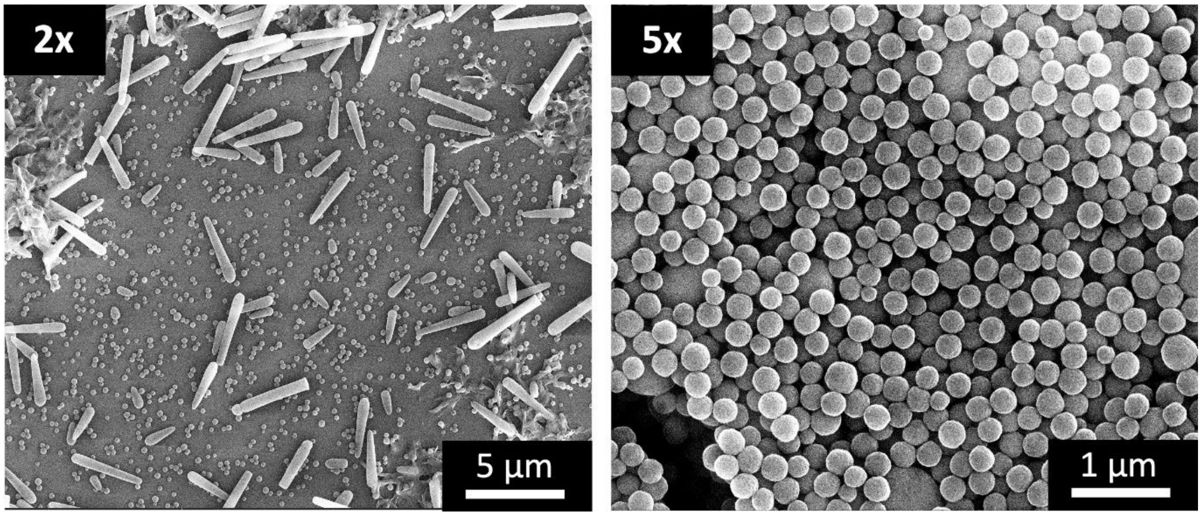


**Figure S3:** Results of unsuccessful upscaling attempts of the one-pot microemulsion synthesis. The amounts of reagents were increased proportionally by factors of two and five. The protocol for synthesis of thick straight fibers was followed with a 1.75:1 (*v*/*v*) ethanol-to-water ratio (see Figure 1, B1/B2). The results were reproducible and demonstrate that the fiber synthesis cannot be easily upscaled. **Left:** Upscaling of the synthesis by a factor of two led to two different particle morphologies: Short rods (~2 µm) and spheres (~0.28 µm). **Right:** Upscaling of the synthesis by a factor of five produced only silica spheres (~0.26 µm).

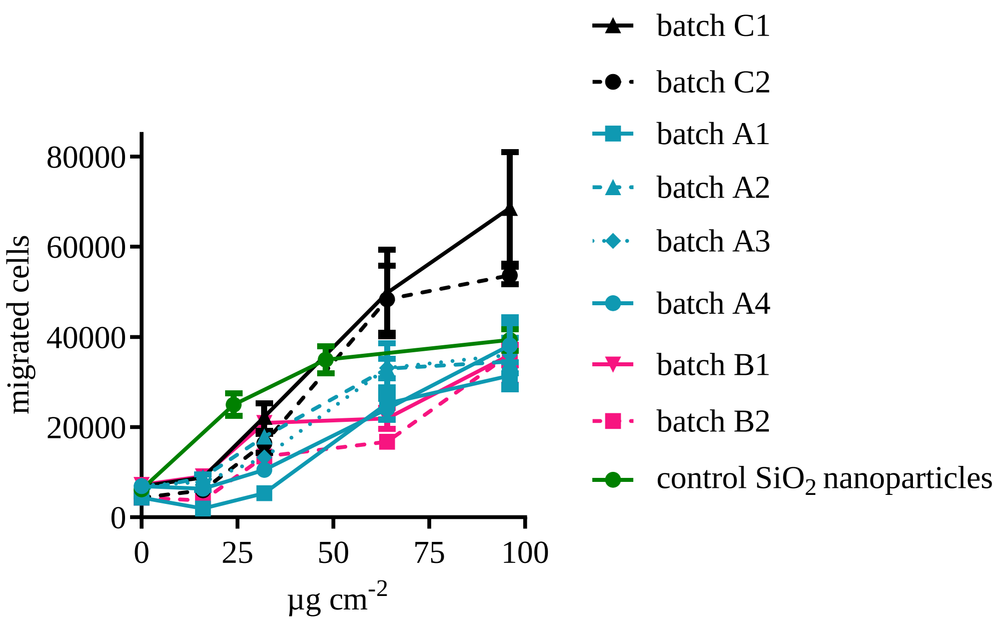


**Figure S4:** Reproducibility of PICMA with silica fibers of different length and thickness. The migration of dHL-60 cells (migrated cells) induced by cell supernatants of nanoparticle-incubated macrophages is plotted against the dose of fibers (in µg cm$^{-2}$). Four batches of straight thin fibers plotted in blue (A1, A2, A3, A4) were grouped as straight thin fibers. Two batches of straight thick fibers (B1, B2) plotted in pink were grouped as straight thick fibers. Two batches of curly thin fibers (C1, C2) plotted in black were grouped as curly fibers. Averages of three independent experiments are shown (*N*=3). Silica nanoparticles served as a control for better comparability.

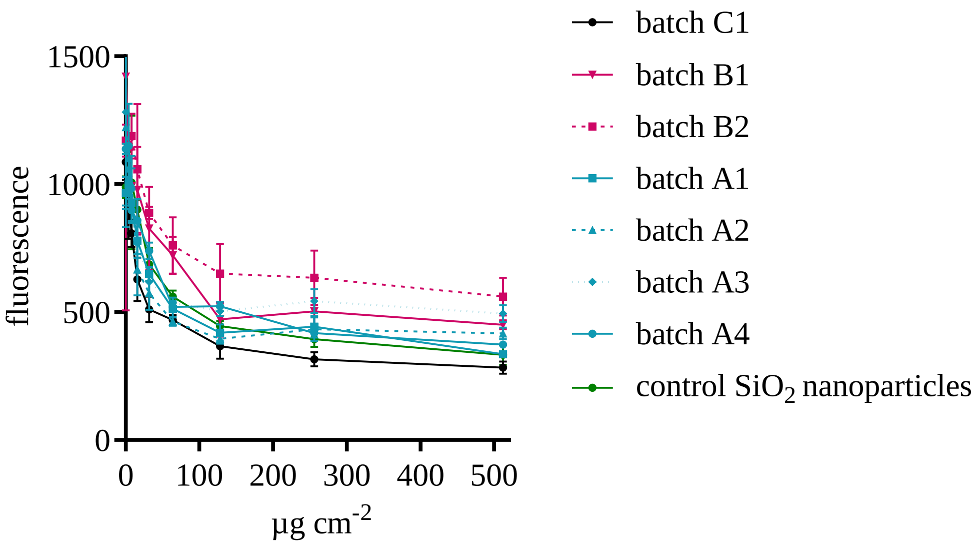


**Figure S5:** Reproducibility of the AlamarBlue Assay with silica fibers of different length and thickness. The fluorescence intensity is plotted against the administered dose of fibers. Four batches of straight thin fibers plotted in blue (A1, A2, A3, A4) were grouped as straight thin fibers. Two batches of straight thick fibers (B1, B2) plotted in pink were grouped as straight thick fibers. One batch of curly thin fibers (C1) is plotted in black. Averages of three independent experiments are shown (*N*=3). Silica nanoparticles served as a control for better comparability.

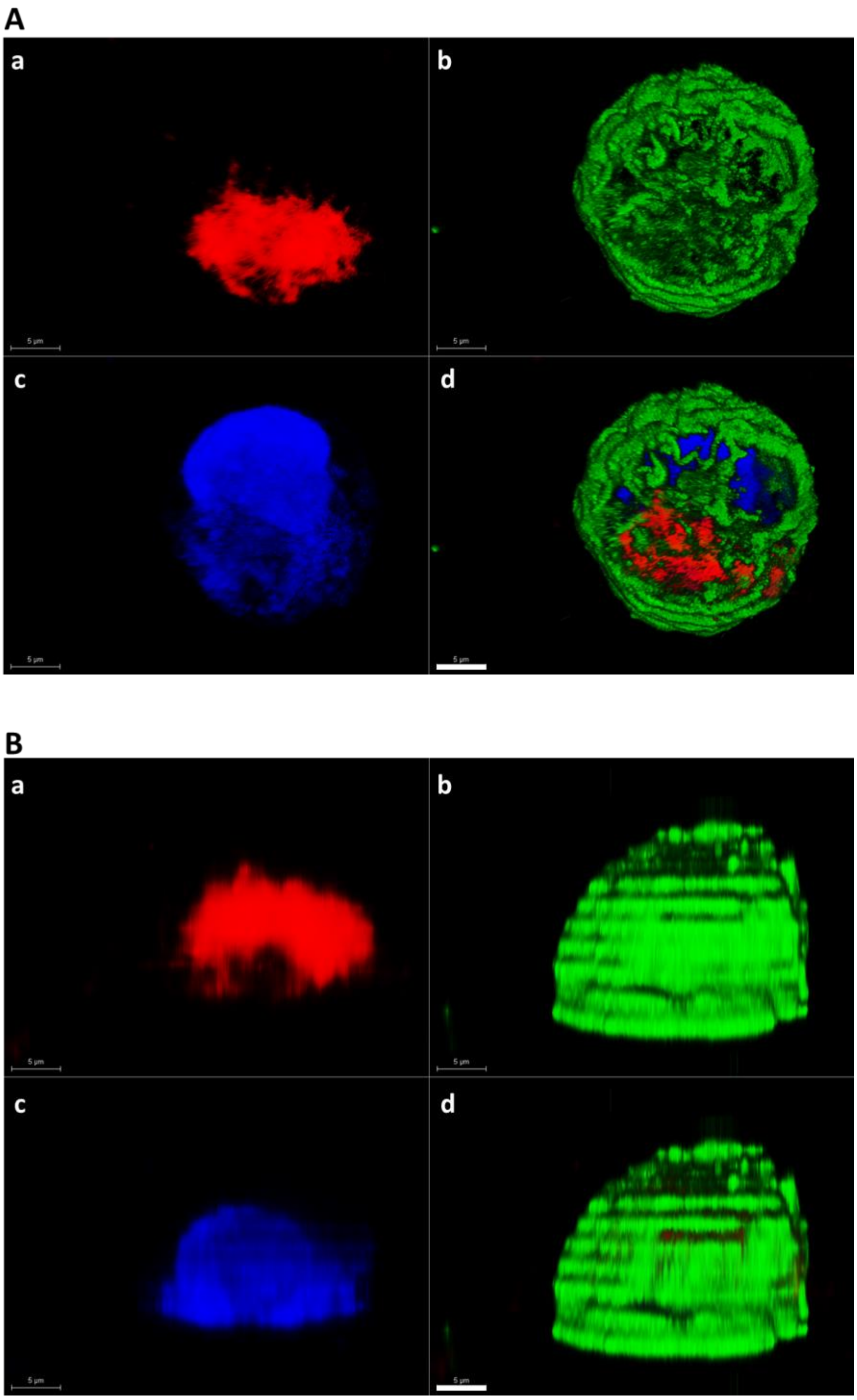


**Figure S6:** Confocal laser scanning microscopy: 3D reconstruction of 12 z-stacks of a THP-1 human macrophage after 24 h incubation with straight thick silica fibers (48 µg $cm^{-2}$). View from the top (**A**), view from the side (**B**). Silica fibers labelled with PEI-rhodamine in red (**a**), actin labelled with AlexaPhalloidin488 in green (**b**), nucleus labelled with Hoechst33342 in blue (**c**), overlay (**d**). Scale bars: 5 µm.
